# Diet-responsive genetic determinants of intestinal colonization in the yeast *Candida albicans*

**DOI:** 10.1128/mbio.02430-25

**Published:** 2025-11-26

**Authors:** Musfirat Shubaita, Mazen Oneissi, Elena Lindemann-Pérez, Cecilia Fadhel Alvarez, Anne-Marie Krachler, Diana M. Proctor, J. Christian Pérez

**Affiliations:** 1 Department of Microbiology and Molecular Genetics, McGovern Medical School, The University of Texas Health Science Center at Houston 12340, Houston, Texas, USA; 2 Graduate School of Biomedical Sciences, The University of Texas MD Anderson Cancer Center UTHealth Houston 4002, Houston, Texas, USA; Yonsei University, Seoul, Republic of Korea

**Keywords:** *Candida albicans*, diet, gut colonization, fatty acids, gut microbiome

## Abstract

Dietary components influence microbial composition in the digestive tract. Although often viewed as energy sources, dietary components are likely to shape microbial determinants of intestinal colonization beyond metabolism. Here, we report that a dietary long-chain fatty acid enhances the yeast *Candida albicans* colonization of the murine gut partly by eliciting modifications to the fungal cell surface. Mice fed an oleic acid-rich diet were readily colonized by *C. albicans* and exhibited higher fungal load in feces compared with rodents fed an isocaloric control diet. Surprisingly, β-oxidation, a catabolic process to break down fatty acids for energy production, was dispensable for *C. albicans* to colonize the high oleic acid diet-fed mice. 16S rRNA analysis detected rather modest differences in the bacterial communities between control and oleic acid-rich diets. We identified *SOK1* as an oleic acid-induced kinase that dictates cell wall mannan exposure and binding to intestinal mucin under anaerobic conditions. Furthermore, oleic acid induced the expression of several *C. albicans* transcription factors that positively regulate intestinal colonization via remodeling of the fungal cell surface. We posit that in environments largely devoid of oxygen like the large intestine, dietary oleic acid favors a *C. albicans* cell surface configuration that enhances gut occupation.

**IMPORTANCE:**

*Candida albicans* is a fungal pathobiont that inhabits the digestive tract of most human adults. The fungus has roles in health and disease because it modulates prominent immune-inflammatory host responses from the gut, and in individuals with debilitated defenses, it can disseminate from the gastrointestinal tract, producing life-threatening infections. Here, we investigate how a dietary component shapes *C. albicans* physiology and ultimately its ability to inhabit the mammalian gut.

## INTRODUCTION

Diet can profoundly impact the microbial composition of the digestive tract. This outcome is thought to reflect the divergent preferences that members of the microbiota exhibit for various dietary components to use as energy sources. For instance, the association of *Prevotella* in the human gut with diets rich in plant-based carbohydrates and fiber (1) can be rationalized by *Prevotella*’s capacity to break down and ferment dietary fiber into short-chain fatty acids. The importance of nutrient acquisition and catabolism for microbes that reside in the mammalian intestine has also been established by large-scale genetic screenings conducted in several gut bacteria to identify determinants of *in vivo* fitness and diet responsiveness (2). However, there are also critical microbial determinants of intestinal colonization beyond nutrient acquisition and metabolism, such as the ability to attach to the intestinal mucosa and withstand host defenses. Whether dietary components also shape these determinants of intestinal colonization remains underexplored.

The yeast *Candida albicans* is a facultative anaerobe that inhabits the digestive tract of humans and other warm-blooded animals (see references 3–5 for recent, comprehensive reviews on *C. albicans* biology in the digestive tract). Despite occasional reports of *C. albicans* isolates found in non-host environments (6), the organism is thought to be vertically transmitted within families (7). This fungal pathobiont modulates prominent immune-inflammatory cascades from the human intestine (e.g*.,* induction of T_H_17 cells) (8–11) and has been associated with several digestive tract-related conditions, including inflammatory bowel disease (10, 12, 13) and liver disease (14, 15). Although fungal traits such as the yeast-to-hyphae transition have extensively been shown to influence *C. albicans* occupation of the mammalian intestine (3, 16–18), our knowledge of fungal adaptations to reside in the gastrointestinal tract remains limited.

The mammalian gut is rich in long-chain fatty acids (LCFAs), which are non-esterified fatty acids 14–20 carbons in length with varying degrees of saturation. Intestinal LCFAs are derived from dietary sources, host cells, and microbial metabolites (19). LCFAs can have diverse effects on microbes. They often serve as nutrients and signaling molecules, although certain unsaturated LCFAs can also exert antimicrobial activity against bacteria such as *Staphylococcus aureus* (20, 21) and *Lactobacillus iners* (22). In yeasts, LCFAs can be catabolized for energy via β-oxidation but also serve as essential building blocks for membrane biosynthesis. The most abundant LCFAs within the intestinal lumen include unsaturated fatty acids such as oleic acid and linoleic acid, and saturated fatty acids like stearic acid and palmitic acid (19).

Here, we investigate the role of a major LCFA, oleic acid, on the biology of *C. albicans*. A rodent diet rich in oleic acid enhanced *C. albicans* colonization of the murine intestine. Because the indigenous microbiota of the animals was maintained in our experiments, we first ruled out that the diet produced major shifts in the bacterial community. Although in aerobic cultures *C. albicans* can break down LCFAs through β-oxidation, this catabolic process was dispensable for the yeast to endure in the digestive tract of mice fed the oleic acid diet. Rather than serving as an energy source, we establish that in anaerobic environments, this LCFA promotes modifications in the cell surface of the organism. Furthermore, we identify oleic acid-induced *C. albicans* regulatory genes that contribute to intestinal colonization. Our findings highlight the importance of the fungal cell wall in interacting with intestinal mucus and how its configuration can be influenced by dietary components.

## RESULTS

### A diet rich in oleic acid promotes *C. albicans* murine gut colonization

The mammalian intestine is rich in long- and short-chain fatty acids. A previous study (23) reported that supplementing a rodent diet with oils rich in 12:0 and 14:0 fatty acids decreased *C. albicans* intestinal colonization, which is consistent with the inhibitory effect on *C. albicans* growth displayed by these molecules *in vitro* (24). Short-chain fatty acids (2–6 carbons) have also been documented to modulate *C. albicans* metabolism, gene expression, and signaling (see reference 25 for a recent review). In contrast to 12:0 or 14:0 fatty acids, the role, if any, that the more common 16- or 18-carbon LCFAs have on the biology of *C. albicans* in the mammalian digestive tract remains unclear. To start addressing this knowledge gap, here, we investigated the effects of the unsaturated LCFA oleic acid (18:1 *cis*-9), which is one of the most abundant LCFAs in nature.

**Fig 1:**
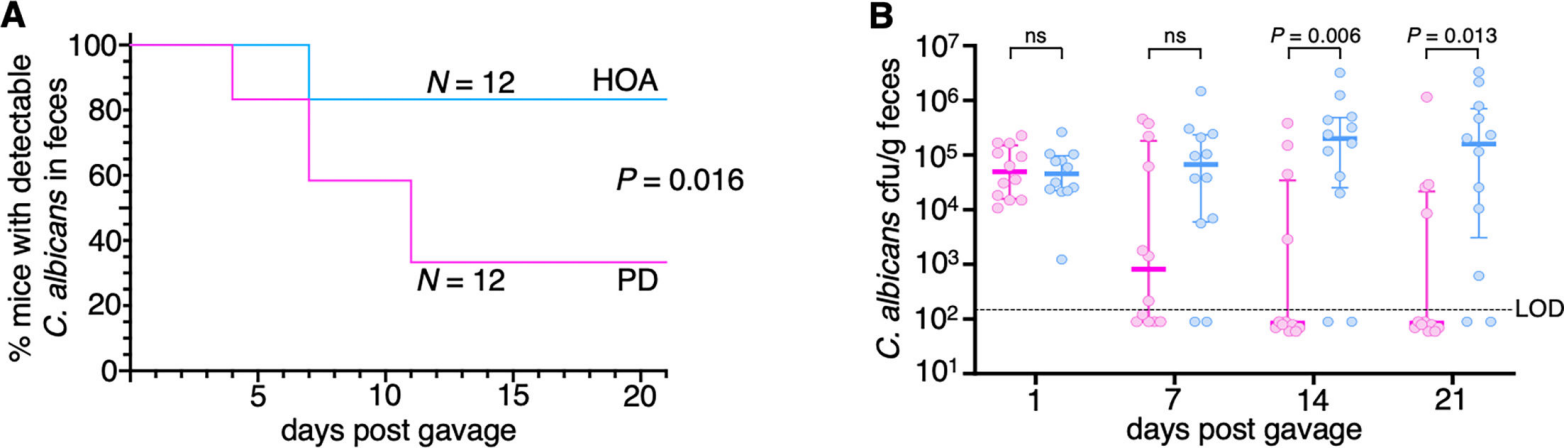
Oleic acid-rich diet promotes *C. albicans* murine gut colonization. (**A**) Swiss Webster mice were fed either the AIN-93G diet (commonly termed “purified diet” and abbreviated PD in this publication, pink) or an isocaloric diet rich in oleic acid (HOA, blue). A week later, *C. albicans* was administered through oral gavage. Plotted is the percentage of mice showing detectable *C. albicans* in feces over time. Fecal pellets were collected and plated twice a week. The experiment was repeated thrice (*N* = 4 animals per diet per experiment), with similar results in each iteration. Shown are the compiled results of all three repeats. Statistical analysis using the log-rank test. (**B**) Quantification of *C. albicans* colony-forming units (cfu) at 1-week intervals in feces of mice included in panel **A**. Thick horizontal lines indicate the median, whereas whiskers span the interquartile range (IQR). Each circle represents an individual mouse. PD and HOA diet in pink and blue, respectively. LOD, limit of detection. Statistical analysis using the Mann-Whitney *U* test. ns, nonsignificant.

Mice were fed either the standard AIN-93G diet (in the literature and herein referred to as “purified diet”) or an isocaloric oleic acid-rich diet, herein termed HOA. The latter contained high-oleic safflower oil (the full composition of the diet can be found in Table S1); 74–79% of the fatty acid composition in this oil is oleic acid. AIN-93G is different from the high fiber chow used in most mouse colonies and was chosen for this study because, in contrast to regular chow, it enables some degree of *C. albicans* gut colonization in mice without antibiotic treatment (26, 27). The animals were left to acclimatize to the diets for 7 days and subsequently were gavaged with *C. albicans*. We quantified the fungal load in feces over time and found that the mice fed the HOA diet were colonized by *C. albicans* to significantly higher levels compared with the animals fed the purified diet (PD) (Fig. 1). This finding suggested that dietary oleic acid may enhance *C. albicans’* ability to inhabit the murine digestive tract.

### Gut bacterial diversity, composition, and community structure differ slightly between mice fed either PD or HOA diet

**Fig 2:**
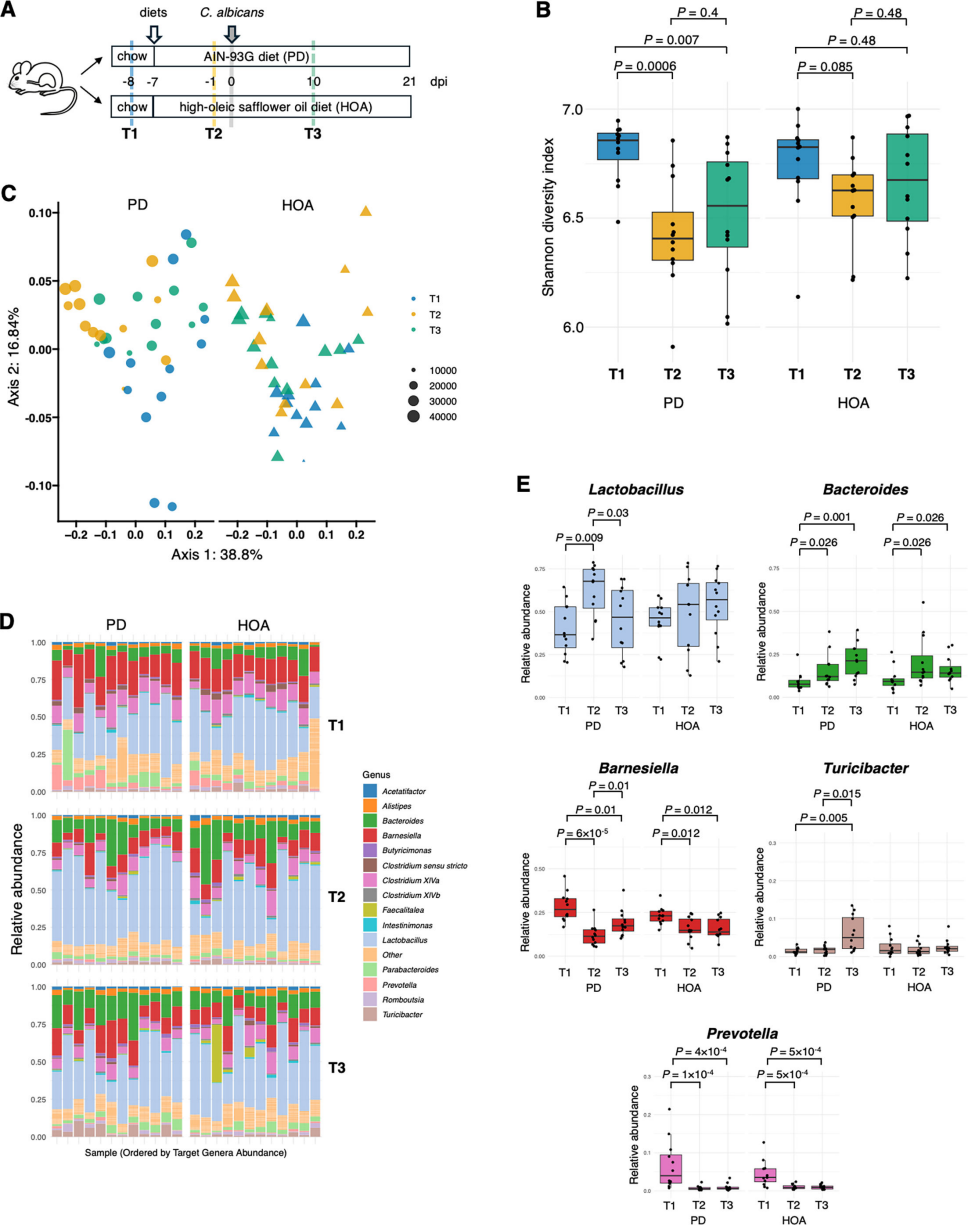
Characterization of the effects of diet on gut bacterial diversity, composition, and community structure. (**A**) Schematic of the study design. Swiss Webster mice were fed either a PD or HOA diet. After 7 days, they were gavaged with *C. albicans*. Fresh fecal pellets were collected at three time points. T1, baseline before introducing either diet; T2, a week after acclimatization to PD and HOA diets but before administering *C. albicans*; T3, ~10 days after *C. albicans* gavage. dpi, day post-infection. (**B**) Boxplot showing the distribution of Shannon entropy across time points. Each panel represents a study arm (PD or HOA). The horizontal line within each box indicates the median, and the box spans the interquartile range (IQR). Statistical analysis using the Wilcoxon rank sum test. *P* values adjusted for multiple comparisons using Benjamini-Hochberg to control the false discovery rate (FDR) at 5%. Between-group comparisons at each time point were also conducted, but no statistically significant differences were detected in any of these comparisons. (**C**) Principal coordinates analysis (PCoA) of weighted UniFrac distances, showing the first two axes. PD and HOA diets are displayed as panels (left and right) to facilitate data visualization. Samples are colored by time point (T1, T2, and T3), with shapes representing diet (circles = PD; triangles = HOA) and point size corresponding to total sequencing depth. (**D**) Relative abundance of the top 15 genera (of 182), with all remaining genera grouped as “other.” Each bar represents one mouse sample, ordered by the combined relative abundance of *Lactobacillus*, *Bacteroides*, and *Barnesiella* from left to right. (**E**) Abundance of five taxa was identified as significantly different across time by three analytical methods. Boxplots follow the same conventions as in panel **B**. Statistical analysis using the Wilcoxon rank sum test. *P* values adjusted for multiple comparisons using Benjamini-Hochberg to control the false discovery rate (FDR) at 5%. *P* values are shown only if comparisons are statistically significant.

Diet changes can alter intestinal bacterial communities. Therefore, we first sought to investigate the effects of the HOA diet on the bacterial microbiome. To assess the bacterial communities, we performed 16S rRNA sequencing of fecal samples collected at various time points (Fig. 2A): baseline before introducing the defined diets (T1), a week after acclimatization to PD and HOA diets but before *C. albicans* gavage (T2), and ~10 days after *C. albicans* administration (T3).

We first examined alpha diversity. Pairwise comparisons of Shannon entropy and other metrics of alpha diversity (Fig. S1A) found no differences between PD and HOA diets at any of the three time points evaluated. There was, however, a significant drop in diversity from T1 (regular chow) to T2 in the PD group (*P* = 0.0006) and a trend toward reduction in the HOA diet (*P* = 0.085) (Fig. 2B). After introducing *C. albicans*, the diversity in the PD group remained low (T3 < T1), whereas in the HOA diet, it remained constant (T3 = T1) (Fig. 2B). Similar trends were observed with additional metrics of alpha diversity (Fig. S1A). Sequencing depth did not significantly vary among groups, indicating that this was not a factor driving the Shannon entropy differences. These findings suggest that although both diets induced early perturbations in microbial diversity, the HOA diet may buffer against long-term disruption.

To investigate changes in community composition, we performed PCoA of the weighted UniFrac distance metric (Fig. 2C). Ordination plots showed separation between T1 relative to T2 and T3, with more pronounced differences for the PD compared with the HOA diet, consistent with alpha diversity metrics. PERMANOVA analysis revealed that when cage effects were not accounted for, diet did not significantly influence microbial composition. However, both time and the time-by-diet interaction were significant, indicating that community structure changed over time in a diet-dependent manner. When the cage was included as a stratification variable in the permutation model, time, diet, and their interaction were statistically significant. Effect sizes were moderate for time (*F* = 4.1) and for the time-by-diet interaction (*F* = 2.8), whereas the effect of diet alone was more modest (*F* = 0.9). Importantly, these shifts in community structure were not attributable to differences in beta dispersion (*P* > 0.1), suggesting that variability between samples did not confound the results. These results highlight the importance of accounting for the non-independence of samples due to cage effects when evaluating the influence of experimental variables.

To determine whether introducing *C. albicans* altered bacterial community composition, we re-ran the PERMANOVA excluding baseline samples (i.e., dropping T1). In this context, time (T3 vs. T2) was no longer a significant factor (*P* = 0.2), indicating that within each diet group, microbial composition remained relatively stable after *C. albicans* gavage. The diet showed a modest effect with a trend toward statistical significance (*P* = 0.08, F = 0.4), indicating that each diet induced a slightly different microbial profile. The interaction between time and diet remained significant, implying that the trajectory of microbiome change differed depending on the diet.

Finally, we examined the taxa contributing to the compositional shifts (Fig. 2D and E; Fig. S1B). Using distance-based redundancy analysis (db-RDA) constrained by time point and diet, we identified the genera most strongly associated with the first two canonical axes (Fig. S1B), which together account for ~17% of the variance. Although some of the most responsive taxa, such as *Prevotella* and *Turicibacter*, were present at low abundance, their temporal trends were still notable (Fig. 2E). More abundant genera, including *Lactobacillus*, *Bacteroides*, and *Barnesiella,* also showed clear and robust shifts over time (Fig. 2E). These genera likely represent biologically meaningful drivers of the observed variation in microbial community structure. It is important to notice, however, that pairwise comparisons of taxa abundance between PD and HOA diets (Fig. S1C) found no statistical differences at any time point, except for *Turicibacter* at T3 (PD > HOA).

Taken together, the findings of the 16S rRNA analysis point to a stabilizing effect of the HOA diet on the microbiome. Nonetheless, only modest differences in bacterial communities were observed between the two diets. Because the magnitude of the changes was rather small, no taxon emerged as a particularly strong candidate to fully account for the effect that the HOA diet had on *C. albicans* gut colonization.

### β-Oxidation is dispensable for *C. albicans* gut colonization in HOA diet-fed mice

We next examined the direct effects that oleic acid can have on *C. albicans* physiology. LCFAs are often catabolized for energy production. In yeasts, fatty acids are broken down through β-oxidation, a process primarily occurring in peroxisomes. The key enzymes of the β-oxidation pathway are well studied in *S. cerevisiae* (28) and conserved in *C. albicans* (29). To determine whether β-oxidation has a role in *C. albicans* gut colonization, we generated a strain in which the *FOX2* gene was deleted (Fig. S2A and B). *FOX2* encodes the second enzyme of the β-oxidation pathway, and null mutations in this gene have been shown to render both *S. cerevisiae* and *C. albicans* β-oxidation deficient (28, 29). As expected, the *fox2* deletion strain was unable to form colonies on agar plates containing oleic acid as the sole carbon source, whereas the complemented add-back strain grew like wild-type (Fig. S2C).

**Fig 3:**
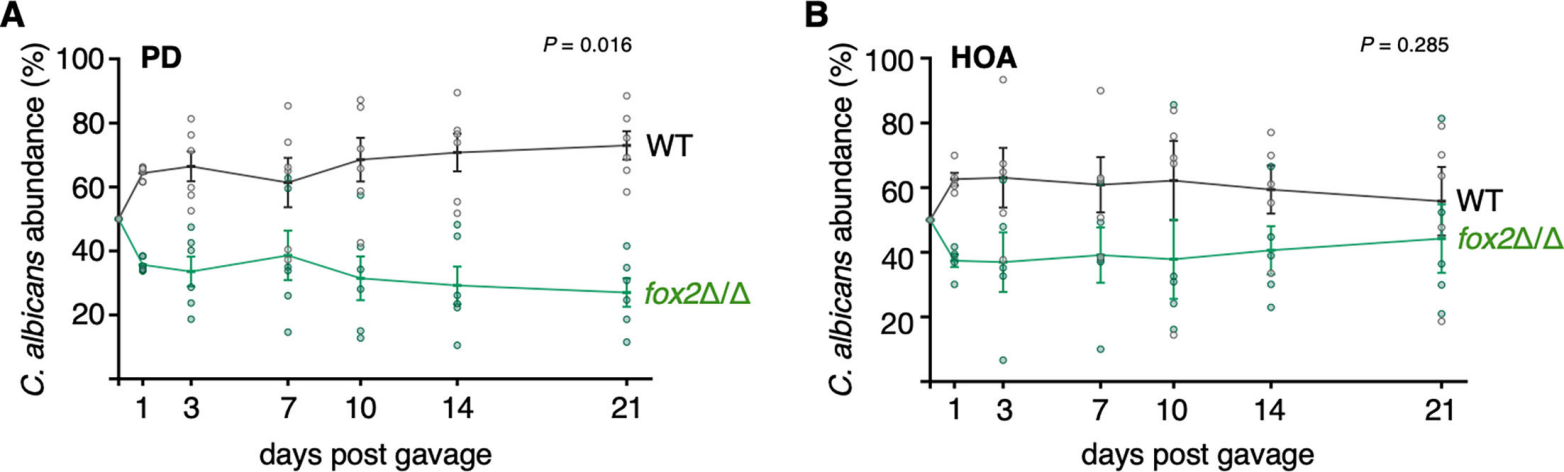
β-Oxidation is dispensable for *C. albicans* to colonize the gut of HOA diet-fed mice. (**A**) PD-fed C57BL/6 mice were gavaged with a 1:1 mixture of wild-type and *fox2*Δ/Δ cells. *C. albicans* colonies were examined in feces at the indicated time points. Mean ± s.e.m. are indicated; each data point represents an individual mouse. (**B**) HOA diet-fed C57BL/6 mice were gavaged with the same strains as in panel A. Statistical analysis (**A and B**) by paired *t*-test (two-tailed).

To probe the competitive fitness of the *fox2* deletion mutant in our mouse model of gut colonization, we gavaged a 1:1 mixture of wild-type and isogenic *fox2* strains and monitored their relative abundance in feces over time. In mice fed the purified diet, the *fox2* mutant displayed a modest but statistically significant reduction in fitness (Fig. 3A). Surprisingly, in the HOA diet, we observed no differences in fitness between the strains (Fig. 3B). These findings indicate that β-oxidation is largely dispensable for *C. albicans* to colonize the digestive tract of mice fed the HOA diet.

### RNA-seq analysis reveals cellular processes and regulatory genes induced by oleic acid under anaerobic conditions

**Fig 4:**
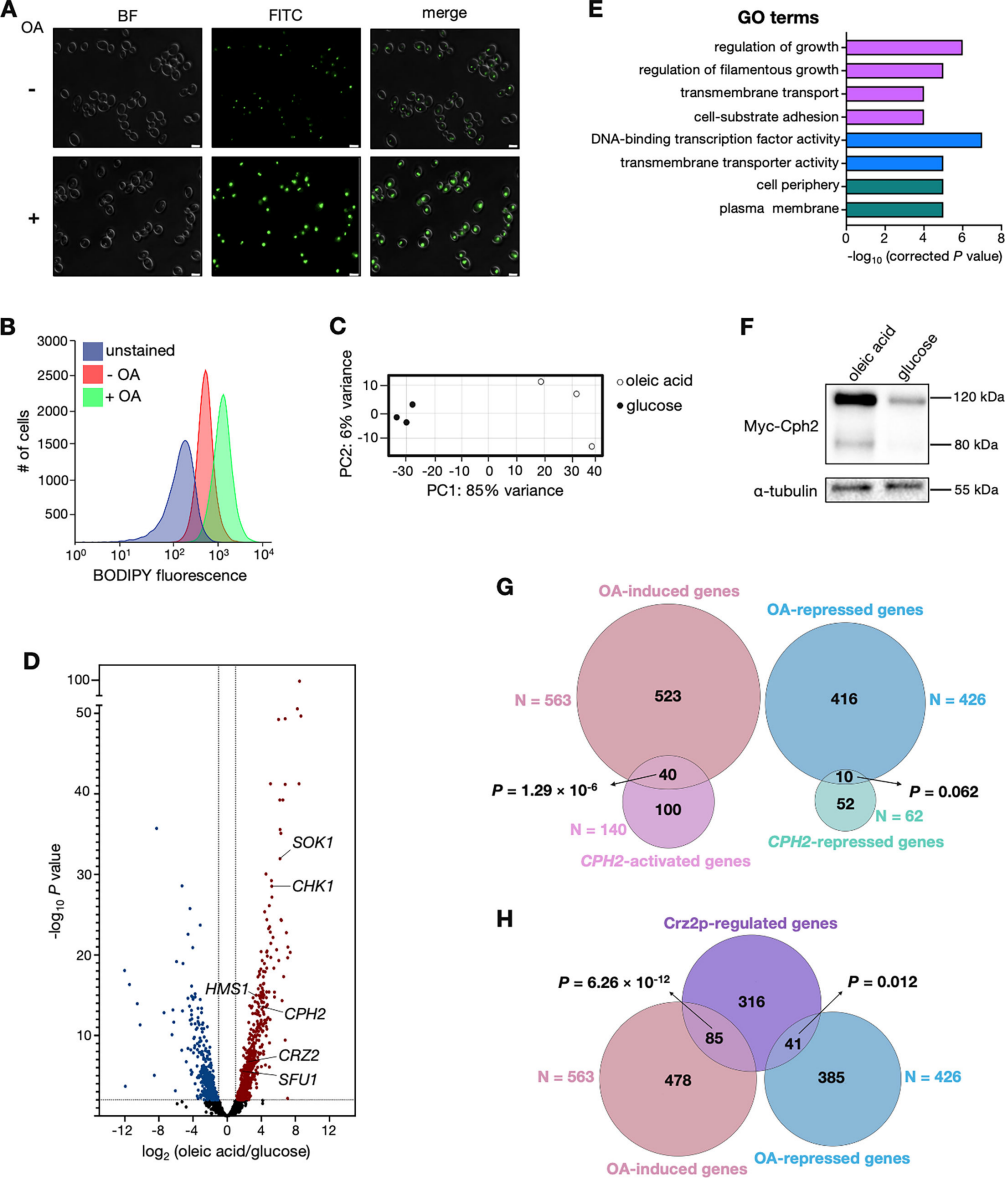
Oleic acid induces the expression of *C. albicans* regulatory genes governing gut colonization. (**A**) Lipid droplet accumulation in wild-type cells incubated under anaerobic conditions in culture medium with or without oleic acid (OA). Lipid droplets were stained with BODIPY. Scale bars, 5 µm. (**B**) Lipid droplet quantification by flow cytometry. Cells were grown as in panel A. (**C**) Principal component analysis (PCA) based on the results of the RNA-seq experiment. Total RNA was prepared from wild-type cells incubated under anaerobic conditions in culture medium containing glucose or oleic acid as the only carbon source. (**D**) Volcano plot showing transcripts upregulated (red) or downregulated (blue) in response to oleic acid. Each dot represents one transcript. (**E**) GO terms enriched in the set of transcripts upregulated in response to oleic acid. Purple, blue, and green indicate “process,” “function,“ and “component“ categories, respectively. (**F**) Western blot analysis of Cph2p. A strain encoding N-terminal Myc-tagged Cph2p was grown as in panel C. Notice that the expected size of the full-length protein is ~120 kDa, whereas its processed form (after cleavage of the ER-embedded C-terminal portion) is ~80 kDa. Alpha tubulin is shown as a loading control. (**G and H**) Venn diagrams showing the overlap between the set of oleic acid-induced genes and *CPH2*-activated or repressed transcripts (**G**) and Crz2p ChIP targets (**H**). *P* values were calculated using the hypergeometric distribution.

Since β-oxidation did not appear to mediate the trait of interest, we next sought to identify by RNA-seq other cellular pathways and components responsive to oleic acid in *C. albicans*. For this experiment, we incubated *C. albicans* in an anaerobic chamber, mimicking the conditions in the large intestine, which is an environment mostly devoid of oxygen. The growth medium was supplemented with 0.2% (7 mM) oleic acid, a concentration within the physiological range of LCFAs in the human intestine, which vary from 1 to 10 mM (30). We established that under these experimental conditions, incubation in oleic acid-containing medium led to the formation of conspicuous intracellular lipid droplets (Fig. 4A and B), as expected. In yeasts, upon uptake or *de novo* synthesis, LCFAs are converted into acyl-CoA molecules, which in turn become incorporated into triacylglycerols (TAGs) within lipid droplets (31, 32).

Our RNA-seq experiment identified 563 protein-coding transcripts upregulated in response to oleic acid and 426 downregulated (compared with dextrose; using the following cutoffs: *P*_adj_ < 0.01 and expression changes > 2-fold) (Fig. 4C and D; Table S2). Gene Ontology (GO) terms enriched in the set of oleic acid-induced transcripts are shown in Fig. 4E, and the most meaningful are followed up below. In the set of oleic acid-repressed transcripts, the enriched GO terms revolved around ribosome function, for example, “ribosome biogenesis” (*P* = 1.05 × 10^−14^) and “structural constituent of ribosome” (*P* = 3.25 × 10^−24^), suggesting limited cellular proliferation under these experimental conditions. This is consistent with the notion that *C. albicans* displays restricted growth in anaerobic environments, as recently reported by others (33), and also observed in our study (Fig. S2D and E).

### Oleic acid promotes the expression of *C. albicans* transcription factors that positively regulate intestinal colonization

The GO term “DNA-binding transcription factor” was one of the most significantly overrepresented in the set of oleic acid-induced genes (Fig. 4E). To explore this lead further, we first asked whether any of the transcription factors (TFs) with the most conspicuous changes in gene expression could be connected to the biology of the fungus in the mammalian digestive tract. As listed in Table 1, of 21 TFs that met a high stringency cutoff (>4-fold induction by oleic acid; *P*_adj_ < 1 × 10^−5^), four (*CPH2*, *HMS1*, *SFU1,* and *CRZ2*) had been implicated in murine gut colonization.

**TABLE 1:** Transcription factors induced by oleic acid under anaerobic conditions^*a*^

Systematic name	Standard name	log_2_(OA/glu)	*P* value	Putative or known regulated trait
*CR_07,530C*	*ECM22*	4.7	1.3 × 10^−20^	
*C2_05,770W*		4.6	4.5 × 10^−21^	
*C2_00,140W*	*NDT80*	4.6	1.1 × 10^−12^	Biofilm formation
*C3_05,930W*	*CTA4*	4.4	4.4 × 10^−26^	Nitrosative stress response
*C5_04,970C*	*PPR1*	4.3	5.2 × 10^−18^	Purine catabolism
*CR_09,960C*	*UGA3*	4.0	3.8 × 10^−21^	Gamma-aminobutyrate utilization
*C5_01,830C*	*HAL9*	4.0	2.1 × 10^−15^	Salt tolerance
*C1_14,340C*	*RIM101*	3.8	1.1 × 10^−18^	Alkaline pH response
*C6_00,280W*	*CPH2*	**3.7**	**2.7 × 10^−14^**	**Intestinal colonization**
*C5_00,670C*	*HMS1*	**3.4**	**1.1 × 10^−15^**	**Intestinal colonization**
*CR_02,560C*	*ASG1*	2.9	2.3 × 10^−14^	
*C1_06,230C*	*MDM34*	2.9	9.8 × 10^−8^	
*C1_01,170C*	*ZCF17*	2.8	1.8 × 10^−6^	
*C1_05,340C*	*ZCF2*	2.7	4.8 × 10^−7^	Response to reactive sulfur species
*C3_05,910W*	*ZCF35*	2.7	5.8 × 10^−11^	
*CR_07,060C*	*CRZ2*	**2.6**	**1.9 × 10^−7^**	**Intestinal colonization**
*C7_04,230W*	*NRG1*	2.3	1.5 × 10^−6^	Negative regulator of filamentation
*CR_06,440C*	*BCR1*	2.3	2.3 × 10^−6^	Adherence and cell surface
*C1_10,020W*	*SFU1*	**2.2**	**3.1 × 10^−6^**	**Iron acquisition; gut colonization**
*C1_12,130C*	*ZCF30*	2.1	4.1 × 10^−6^	
*CR_07,450C*	*MNL1*	2.1	5.2 × 10^−7^	Adaptation to weak acid stress

^*a*^ OA, oleic acid; glu, glucose. Boldface entries are discussed in the text.

*CPH2* and *HMS1* form a regulatory cascade that controls the expression of cell surface components and prevents filamentation under anaerobic conditions (34). Deletion of either gene reduces *C. albicans* fitness in the standard murine gut colonization model (35, 36). We carried out western blot analysis of Cph2p to validate the RNA-seq finding (Fig. 4F). Indeed, the protein levels of Cph2 were conspicuously higher in cells incubated in oleic acid-containing medium, consistent with its up-regulation at the transcript level. Cph2 is an endoplasmic reticulum-bound protein that undergoes cleavage to release its active form (37). In our western blot analysis, in addition to the full-length protein (~120 kDa including the tag), we detected the cleaved product (~80 kDa) in cells cultured in oleic acid-containing media. Furthermore, we established that there was a modest but statistically significant overlap between the set of *CPH2*-regulated genes and oleic acid-induced transcripts (Fig. 4G). Although these data sets come from experiments carried out in different culture conditions, the overlap is consistent with the notion that *CPH2* may, at least in part, mediate the oleic acid response in *C. albicans*.

*CRZ2* and *SFU1* were the other oleic acid-induced TFs that have been shown to contribute to *C. albicans* gut colonization (38, 39). The latter encodes a major transcriptional repressor of iron uptake genes. From nine direct targets of Sfu1p regulation identified by chromatin immunoprecipitation (38), we found that the expression of five of them was influenced by oleic acid. Crz2p, on the other hand, is induced under low oxygen conditions and controls the expression of cell wall components (39). We found a statistically significant overlap between the set of Crz2p-bound target genes (defined by chromatin immunoprecipitation) and oleic acid-induced transcripts (Fig. 4H). Although the data sets included in these comparisons are derived from experiments carried out in different culture conditions, the overlaps are consistent with the idea that *CRZ2*, and to a lesser extent *SFU1*, may also have a role (albeit minor) in the response to oleic acid.

### *SOK1* is an oleic acid-induced kinase that promotes yeast cell adherence and binding to intestinal mucin under anaerobic conditions

**Fig 5:**
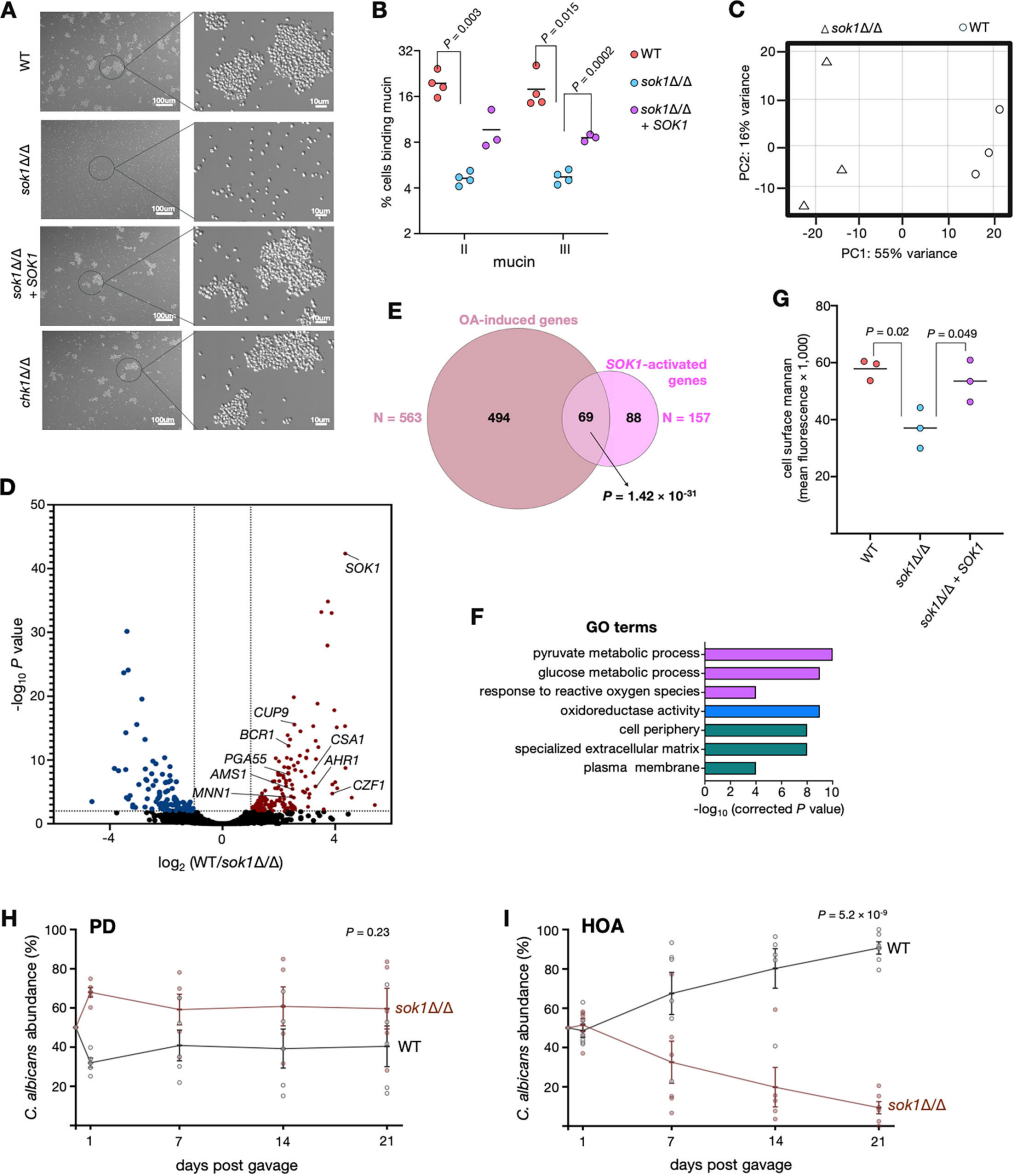
The oleic acid-induced kinase *SOK1* promotes binding to intestinal mucin and intestinal colonization in HOA diet-fed mice. (**A**) *SOK1*-dependent yeast cell aggregation. The indicated strains were incubated under anaerobic conditions for 72 h in culture medium containing oleic acid and examined by microscopy. Representative images are shown. Insets are enlarged to the right. (**B**) *C. albicans* binding to mucin is *SOK1*-dependent. Fluorescently labeled mucin type II and III were incubated with the indicated *C. albicans* strains. Flow cytometry was used for quantification. In total, 30,000 single cells were examined per strain per experiment. Each dot represents an independent experiment. Statistical analysis using Welch’s *t*-test. (**C**) Principal component analysis (PCA) based on the results of the RNA-seq experiment. Total RNA was prepared from wild-type and *sok1*Δ/Δ cells incubated under anaerobic conditions in culture medium containing oleic acid. (**D**) Volcano plot showing transcripts up- (blue) or down-regulated (red) in *sok1*Δ/Δ cells. Each dot represents one transcript. (**E**) Venn diagrams showing the overlap between the set of oleic acid-induced genes and transcripts positively regulated by *SOK1* (downregulated in *sok1*Δ/Δ cells). *P*-value was calculated using the hypergeometric distribution. (**F**) GO terms enriched in the set of transcripts positively regulated by *SOK1* (downregulated in *sok1*Δ/Δ cells). Purple, blue, and green indicate “process,” “function,” and “component” categories, respectively. (**G**) *sok1*Δ/Δ cells exhibit decreased cell surface mannan. Concanavalin A conjugated to Alexa Fluor 488 was incubated with the indicated *C. albicans* strains. Flow cytometry was used for quantification. In total, 30,000 single cells were examined per strain per experiment. Each dot represents the mean fluorescence of an independent experiment. Statistical analysis by Welch’s *t*-test. (**H and I**) Colonization fitness of the *sok1*Δ/Δ strain. C57BL/6 mice were fed PD or HOA diets and gavaged with a 1:1 mixture of wild-type and *sok1*Δ/Δ cells. *C. albicans* colonies were examined in feces at the indicated time points. Mean ± s.e.m. are indicated; each data point represents an individual mouse. Statistical analysis by paired *t*-test (two-tailed).

The overrepresentation of the GO terms “regulation of filamentous growth” and “cell-substrate adhesion” in the set of oleic acid-induced genes prompted us to evaluate the anaerobic *C. albicans* cultures by microscopy. We established that in oleic acid-containing media, the fungal cells maintained the yeast (blastospore) morphology, with minimal—if any—evidence of filamentation (Fig. S3A). The yeast state persisted even after 3–5 days of incubation in the anaerobic chamber. We also found that *C. albicans* formed cell aggregates upon extended incubation under these experimental conditions (Fig. 5A).

Because the cell aggregation phenotype observed under anaerobic conditions could reflect a “sticky” cell surface to adhere to other biologically relevant substrates, we were interested in identifying oleic acid-induced signaling or regulatory genes mediating this phenotype. The most strongly induced transcripts (>32-fold induction by oleic acid; *P*_adj_ < 1 × 10^−25^; 17 genes passed these thresholds) included only three genes in the category of signaling or regulatory functions. All three encoded kinases: *TPK1*, a cAMP-dependent protein kinase subunit involved in filamentation regulation (40); *CHK1*, a histidine kinase controlling cell wall properties (41); and *SOK1*, a less understood kinase with reported roles in response to farnesol (42) and biofilm vertical extension (43). Since filamentation did not appear to play any role under our experimental conditions, we focused our genetic analysis on *CHK1* and *SOK1*. We probed the *chk1* and *sok1* deletion strains for their ability to form cell aggregates under anaerobic conditions and found that this phenotype was dependent on *SOK1* but not on *CHK1* (Fig. 5A). Reinserting a wild-type copy of *SOK1* into the homozygous *sok1* deletion strain restored near wild-type levels of aggregation, indicating that the phenotype was indeed dependent on this gene (Fig. 5A).

We next sought to probe the adherence of the wild-type and *sok1* deletion strains to a biologically relevant substrate. The intestinal epithelium is covered with mucus, a layer that serves as a habitat for the gut microbiota, including *C. albicans* (44, 45). Thus, we tested the binding of the fungal cells to gut mucin. To do this, we fluorescently labeled mucin and incubated this preparation with *C. albicans* cells grown under anaerobic conditions in oleic acid-containing media. Remarkably, oleic acid promoted mucin binding to fungal cells (Fig. S3B). Notably, although the wild-type reference strain showed robust binding to mucin, the *sok1* deletion mutant exhibited a significant reduction in binding (Fig. 5B). From these experiments, we conclude that under anaerobic conditions, the oleic acid-induced kinase *SOK1* promotes yeast cell adherence and binding to gut mucin.

### *SOK1* regulates mannan exposure under anaerobic conditions

To identify molecular functions and/or cellular components regulated by the *SOK1* kinase in *C. albicans*, we conducted an RNA-seq experiment comparing the transcriptome of the wild-type reference strain with that of an isogenic *sok1* deletion mutant (Fig. 5C). To the best of our knowledge, no transcriptome data have been reported for this gene. The RNA-seq experiment was conducted with cells incubated in oleic acid-containing medium under anaerobic conditions, as the *SOK1* transcript is upregulated in these conditions. We identified 257 protein-coding transcripts with altered expression in the *sok1* mutant (157 down- and 100 up-regulated compared with the wild-type reference strain using the following cutoffs: *P*_adj_ < 0.01 and expression changes > 2-fold (Fig. 5D; Table S3). To determine whether *SOK1* is indeed a mediator of the oleic acid response, we compared the *sok1* transcriptome data to the set of oleic acid-regulated transcripts. We found that almost half (69 of 157) of the *SOK1*-activated transcripts corresponded to oleic acid-induced genes (*P* = 1.42 × 10^−31^), indicating that a significant proportion of the gene expression changes in response to oleic acid is likely dependent on *SOK1* (Fig. 5E).

The GO terms enriched in the set of genes displaying *sok1*-dependent expression (Fig. 5F) suggested that the *SOK1* kinase may influence multiple cellular functions and components in *C. albicans*, including oxidoreductase activity, metabolism, and cell periphery. However, exclusively in the context of the oleic acid response, that is, considering only the overlap between *SOK1*-activated transcripts and oleic acid-induced genes, the top GO term was “cell periphery” (*P* = 1.29 × 10^−5^). Indeed, the expression of genes encoding prominent cell wall proteins such as *CSA1* and *PGA55*; transcription factors linked to adhesion and cell wall integrity, including *BCR1* (46)*, CZF1* (47)*, CUP9* (48), and *AHR1* (49); and enzymes that catalyze mannan modifications, including the alpha-1,3-mannosyltransferase *MNN1* and the alpha-mannosidase *AMS1,* were all dependent on *SOK1*.

The outermost layer of the *C. albicans* cell wall is primarily composed of mannan. We reasoned that because of its location on the surface of the fungal cell, this layer is likely to interact with intestinal mucin. Because the *sok1* deletion strain exhibited reduced binding to mucin (Fig. 5B), we hypothesized that this may be connected to *sok1*-dependent alterations in the mannan layer. To test this idea, we probed the overall exposed mannan on *C. albicans* wild-type and *sok1* mutant cells by staining with fluorescently labeled concavalin A (ConA), a lectin known for its high affinity for α-D-mannosyl and α-D-glucosyl residues, which are key mannan components. Although cell surface mannan was slightly lower in oleic acid compared with glucose medium (Fig. S3C), the *sok1* deletion strain showed a significant decrease in ConA binding compared with the wild-type reference strain, indicating a reduction in exposed mannan (Fig. 5G). Taken together, these results implicate *SOK1* in the regulation of mannan exposure on the *C. albicans* cell surface.

### *SOK1* contributes to intestinal colonization in HOA diet-fed mice

Since *C. albicans* binding to intestinal mucin was *SOK1*-dependent (Fig. 5B) and the expression of this kinase was strongly induced by oleic acid (Fig. 4D), we reasoned that this gene may be required for the fungus to colonize the murine intestine, particularly in animals fed the HOA diet. To test this hypothesis, we gavaged a 1:1 mixture of wild-type and isogenic *sok1* strains in mice fed either diet and monitored the relative abundance of both strains in feces over time. In mice fed the purified diet, both strains displayed comparable fitness (Fig. 5H). By contrast, the *sok1* strain exhibited a significant reduction in fitness in mice fed the HOA diet (Fig. 5I) (Fungal load in absolute numbers is plotted in Fig. S3D). These results establish *SOK1* as a genetic determinant of *C. albicans* intestinal colonization and indicate that the described connection between fatty acid and colonization-associated traits is physiologically relevant.

### Effect of other monounsaturated fatty acids

**Fig 6:**
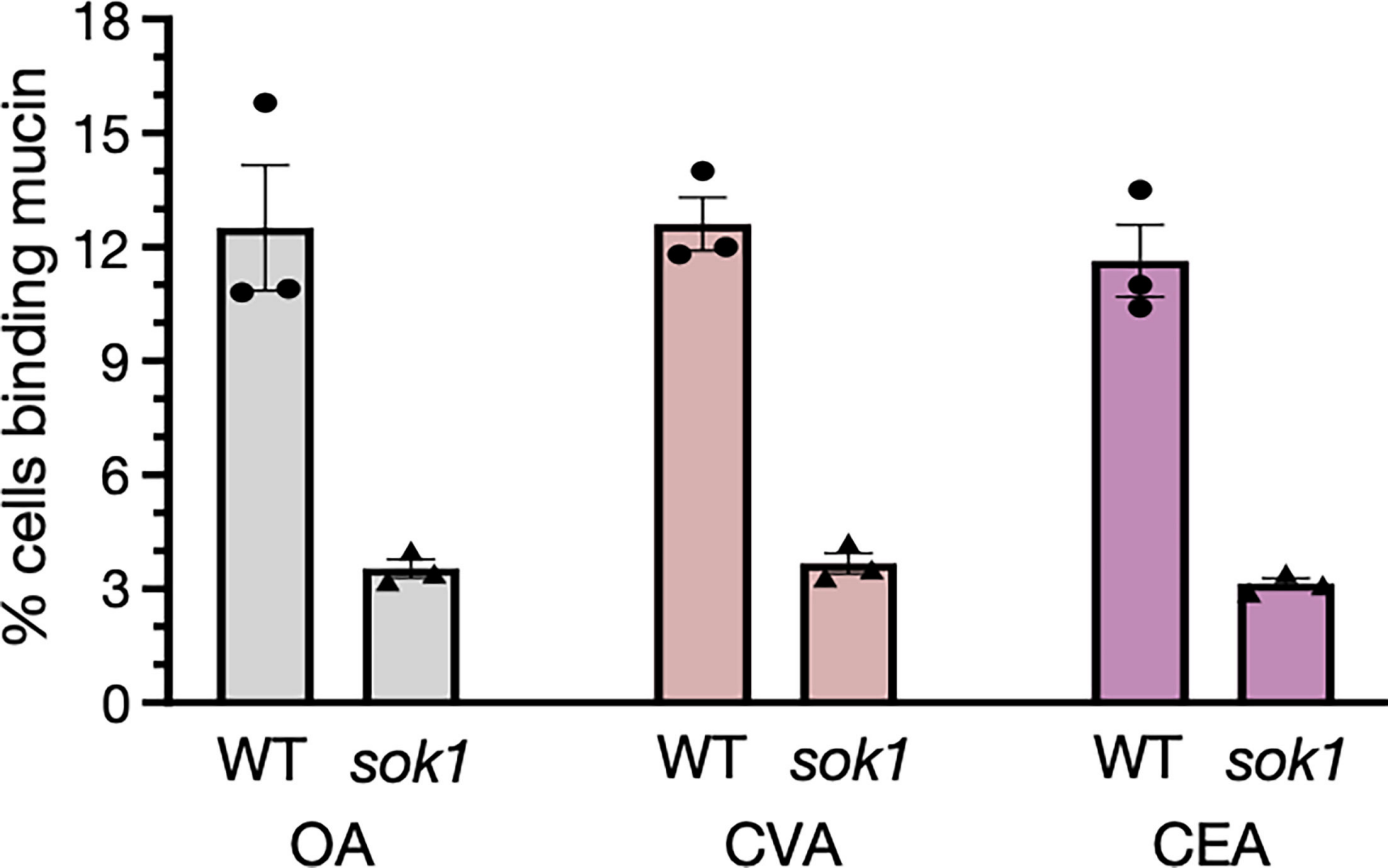
Effect of other monounsaturated fatty acids on *C. albicans* binding to mucin. Fluorescein-labeled mucin type II was incubated with the indicated *C. albicans* strains. Cells were grown anaerobically in YNB broth supplemented with one of three fatty acids: oleic acid (OA), *cis*-vaccenic acid (CVA), or *cis*-11-eicosenoic acid (CEA). Flow cytometry was used for quantification. In total, 30,000 single cells were examined per strain per experiment. Each dot represents an independent experiment. Plotted are mean ± s.e.m.

Oleic acid is a monounsaturated fatty acid. To address whether other monounsaturated fatty acids could also affect *C. albicans* cell physiology in a similar manner to oleic acid, we evaluated the ability of wild-type and *sok1* mutant cells to bind mucin after incubation in media supplemented with *cis*-vaccenic acid (18-carbon chain) or *cis*-11-eicosenoic acid (20-carbon chain). As shown in Fig. 6, both fatty acids produced effects comparable with oleic acid. Although a larger and more structurally diverse set of LCFAs must be probed to stringently examine the specificity of the effects, this result suggests that several long-chain monounsaturated fatty acids may elicit similar responses in *C. albicans*.

## DISCUSSION

In this communication, we have identified the LCFA oleic acid as an important dietary component that shapes *C. albicans* intestinal colonization. Rather than serving primarily as an energy source for the fungus, we report that under anaerobic conditions, this fatty acid activates regulatory pathways linked to the *C. albicans* cell surface, enhancing traits associated with gut colonization. Several results support this notion. First, mice fed a diet rich in oleic acid displayed heightened levels of *C. albicans* gut colonization (Fig. 1); second, β-oxidation was dispensable for the fungus to colonize the intestine of the oleic acid diet-fed mice (Fig. 3); third, transcripts encoding cell wall components and regulators were upregulated in response to the fatty acid (Fig. 4); and fourth, Sok1, a major oleic acid-induced putative kinase, mediated cell wall mannan exposure and binding to intestinal mucin (Fig. 5). Adhesion and binding to intestinal mucin are critical traits for gut microbes (50–52) and likely depend on the cell surface structure adopted by the fungus. Thus, oleic acid-induced alterations to the cell wall structure can significantly influence *C. albicans* persistence in the mammalian gut.

**Fig 7:**
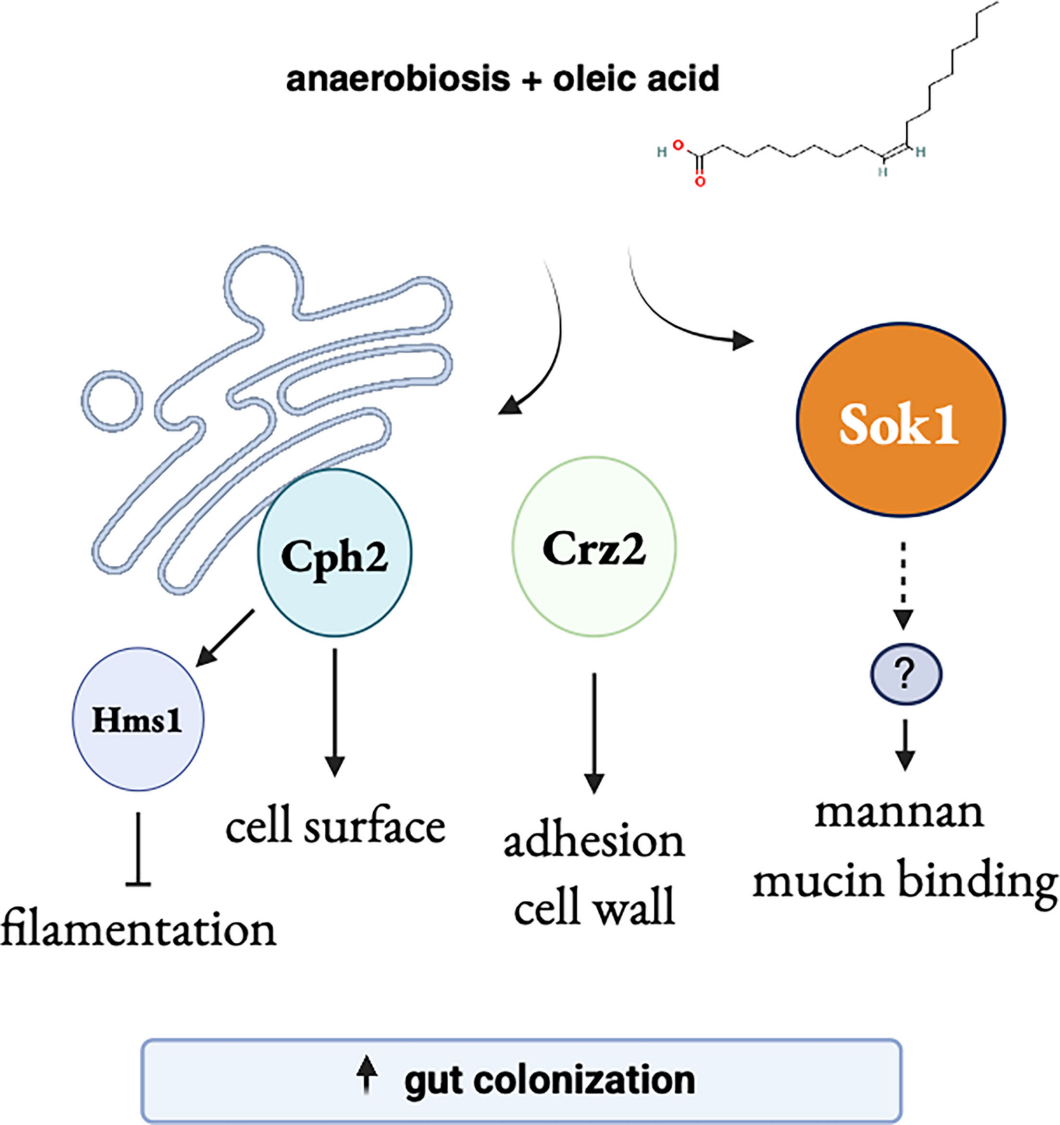
Cartoon depicting regulatory proteins implicated in oleic acid response. In anaerobic environments, oleic acid induces the expression of the transcription factors Cph2, Hms1, and Crz2 as well as the kinase Sok1. These regulatory proteins govern multiple cell surface-related properties that ultimately contribute to *C. albicans* fitness in the murine gut. A yet-to-be-identified signaling pathway and/or transcription factor(s) may lie downstream of Sok1. Created in BioRender (https://BioRender.com/zce99oo).

We have identified *SOK1*, a gene that encodes a putative kinase, as a novel genetic determinant of *C. albicans* gut colonization and an important mediator of the response of this yeast to oleic acid (Fig. 7). Almost half of the transcripts downregulated in the *sok1* deletion strain (i.e., positively regulated by *SOK1*) were also induced by oleic acid. Salient GO terms in the set of genes exhibiting *SOK1*-dependent expression included “cell periphery,” which is consistent with the reduction in mannan that we observed in the *sok1* deletion mutant. Mannan is a polysaccharide that makes up the outermost layer of the fungal cell wall. It seems reasonable to assume, therefore, that this reduction in mannan underlies, at least in part, the adhesion and mucin binding phenotypes of the *sok1* strain. Besides the amount of mannan, the exact configuration and structure of the polymer (monomer composition, linkages, and branching patterns) likely influence binding to mucin. This may explain the observation that, in wild-type cells, oleic acid promoted mucin binding (Fig. S3B) despite slightly lowering the overall cell surface mannan levels (Fig. S3C). *SOK1* has been shown to have a role in *C. albicans* biofilm vertical extension (43) and degradation of the Nrg1 protein, a repressor of hyphal development (42). Our findings now implicate *SOK1* in intestinal colonization; specifically, we demonstrate that *SOK1* is a key intermediary between fatty acid response and cell wall remodeling.

How does oleic acid affect *C. albicans* cell physiology? Our data indicate that in anaerobic environments containing oleic acid (at physiological levels), the yeast accumulates conspicuous lipid droplets in its cytoplasm. In the model yeast *S. cerevisiae*, lipids stored in this organelle are either catabolized for energy or used for membrane biosynthesis. Our observations suggest that under the experimental conditions evaluated, LCFA catabolism via β-oxidation is limited. Therefore, we speculate that oleic acid may primarily impact cell membrane composition. In this scenario, the activation of regulatory circuits such as *CPH2* and *CRZ2*, which control the expression of cell surface components, may be triggered by changes in cell membrane composition and/or fluidity. It is well established that membrane fluidity can substantially vary depending on the degree of saturation of its fatty acid chains (53, 54). In agreement with this idea, other monounsaturated LCFAs had a similar effect on *C. albicans* mucin binding (Fig. 6). Another possibility is that oleic acid or its derivatives act as signaling lipids, regulating specific intracellular pathways as certain fatty acid derivatives do in higher eukaryotes (55). Further investigations are needed to distinguish these alternative models.

Our analysis of the fecal bacterial microbiome in mice fed a PD or an oleic acid-rich diet suggests that the latter had a stabilizing effect on the microbial community. The largest shift in bacterial composition occurred in the transition from chow to defined diets (particularly to the purified diet), reflecting major but well-established differences in their formulation (56). Accordingly, the largest shifts in microbial community structure occurred between T1 (chow) and T2, as shown in Fig. 2C, and were accompanied by corresponding changes in taxon abundances (Fig. 2E). *Turicibacter* was the only taxon that exhibited a significant difference in abundance between PD and HOA diets (Fig. S1C), an outcome detected after *C. albicans* gavage (i.e., at T3). *Turicibacter* was more abundant in PD-fed mice, the group displaying lower levels of *C. albicans* colonization. This negative correlation between *Turicibacter* and *C. albicans* is consistent with previous observations in the murine intestine (57) and in older adult humans (58), raising the possibility that the taxa may antagonize each other to some extent. Beyond differences in microbial taxa, a change in the metabolic milieu of the murine intestine in response to oleic acid is also plausible but was not addressed in this study.

Although our results indicate that oleic acid exerts direct effects on *C. albicans* physiology, enhancing gut colonization, we cannot rule out that dietary oleic acid can modulate additional traits that contribute to colonization of the murine digestive tract. For example, this fatty acid has anti-inflammatory activity on the host (59); therefore, its presence in the diet may dampen immune-driven mechanisms to clear the fungus. Furthermore, from our *C. albicans* transcriptome data, it is clear that in addition to the cell wall, other cellular functions and processes are also influenced by oleic acid. In fact, the regulatory and signaling genes that we highlight in this report (*CPH2*, *CRZ2,* and *SOK1*) can explain no more than half of the transcripts upregulated in response to oleic acid (based on the overlaps in regulated genes). Therefore, additional oleic acid-induced changes in *C. albicans* physiology may also shape fitness in the murine digestive tract. It is also worth pointing out that the findings reported here are in the SC5314 background; other *C. albicans* isolates may differ in how they respond to the presence of fatty acids and in the particular regulatory genes involved because of widespread diversification in transcription factor function across strains (60–62).

Oleic acid is a common constituent of many foods regularly consumed by humans. Dietary oleic acid, however, has also been shown to induce obesogenic hyperplasia at physiologic levels (63). Furthermore, plasma monounsaturated fatty acids, which are mostly oleic acid, are associated with human obesity (63). Intestinal mycobiota studies in humans have found a higher prevalence of *C. albicans* in obese individuals (64), an observation that could be explained by the link reported here between fatty acid and this fungus. High levels of intestinal oleic acid can be caused by the malabsorption of fats and bile acids (65). This condition can be seen in inflammatory bowel disease. For example, Crohn’s disease can damage the lining of the small intestine, specifically the ileum, which is crucial for absorbing fats and bile. *C. albicans* has also been associated with inflammatory bowel disease, although in complex ways (10, 12, 13).

Our findings highlight the notion that dietary components shape microbial traits—other than catabolism—that enhance persistence in the digestive tract. For the human pathobiont *C. albicans*, these traits include adhesion to intestinal mucin and cell surface composition, underscoring the importance of the fungal cell wall to inhabit the intestinal niche.

## MATERIALS AND METHODS

### Strains, media, and anaerobic conditions

All *C. albicans* strains used in this study are listed in Table S4 and are derivatives of clinical isolate SC5314 (66). The *fox2*Δ/Δ strain was constructed as described (67) using the *C. albicans* LEUpOUT CRISPR system. The complemented strain was generated by integrating the *FOX2* open reading frame into its native locus. dTomato-expressing strains were constructed by inserting the NotI-linearized pENO1-dTom-NATr plasmid (68), which harbors a codon-optimized dTomato gene under control of the constitutive *ENO1* promoter. Oligonucleotides employed for strain construction are listed in Table S5. The *sok1*Δ/Δ and *chk1*Δ/Δ strains, as well as their respective add-backs, were kindly provided by J. Morschhäuser (Universität Würzburg, Germany) and are part of their kinase deletion collection (69). The strain encoding epitope-tagged *CPH2* was kindly provided by H. Liu (University of California, Irvine, CA, USA) and has been described (37). The *C. albicans* strains were routinely propagated in YPD medium (1% yeast extract, 2% peptone, and 2% dextrose) at 30°C. Transformants were selected on YPD agar plates containing nourseothricin.

Oleic acid (Sigma, Cat. No. 75090) was added to yeast nitrogen base (YNB) broth (with ammonium sulfate, MP Biomedicals) or Todd-Hewitt broth (THB) to a final concentration of 0.2% (7 mM). Tween 80 (Sigma, Cat. No. P1754) was added to a final concentration of 2% to increase oleic acid solubility. Control YNB or THB media (i.e., containing dextrose instead of oleic acid) were also supplemented with the same Tween 80 concentration. Anaerobic cultures were carried out in an anaerobic workstation (Baker’s Bugbox Ax) with a gas mixture consisting of 80% nitrogen, 10% carbon dioxide, and 10% hydrogen. For all experiments conducted under anaerobic conditions, the culture media were preincubated in the anaerobic workstation for at least 24 hours before *C. albicans* inoculation to eliminate trace amounts of oxygen.

The *C. albicans* cultures to evaluate the effect of fatty acids were seeded as follows. To reach high density, strains were first grown overnight aerobically in liquid YPD. Overnight YPD cultures were washed twice with 1× phosphate-buffered saline (PBS). Subsequently, washed cell suspensions were brought into the anaerobic workstation to inoculate (1:50 dilution) into either YNB broth or THB, which had been preincubated in the anaerobic environment.

### Mouse gut colonization

Animals were housed at 22°C, ambient humidity, and with a 12  h light-dark cycle.

Female SPF Swiss Webster mice (Taconic) at 5–8 weeks of age were randomly assigned to two groups and fed either PD or HOA diet (both purchased from Research Diets, Inc). Animals were left to acclimate to the diets for 7 days. Subsequently, they were gavaged with 1 × 10^7^ *C. albicans* cells as described (16, 35). Fecal pellets were collected at the indicated time points and immediately frozen for microbiome analysis or processed for fungal load determination by plating serial dilutions on yeast mold agar (supplemented with chloramphenicol and kanamycin to prevent bacterial growth).

To assess colonization fitness of the *fox2* and *sok1* mutant strains, a 1:1 mixture of dTomato-expressing reference strain and isogenic deletion mutant (total inoculum = 2 × 10^7^ *C. albicans* cells) was gavaged in female C57BL/6 mice (The Jackson Laboratory) that had been fed either the PD or the HOA diet. The proportion of each strain was determined at the indicated time points by plating fecal pellets on yeast mold agar supplemented with chloramphenicol and kanamycin. The animal experiments were repeated at least twice.

### Microbiome analysis

Fresh fecal pellets were collected and kept frozen (−80°C) until DNA extraction. The ZymoBiomics DNA Microprep Kit (Zymo Research) was used to isolate DNA following the manufacturer’s instructions; 16S rRNA gene sequencing was performed by Novogene following their standard operating procedures. Briefly, the V3–V4 region was PCR amplified using barcoded oligos 341F and 806R. Equimolar concentrations of each sample were pooled and sequenced on an Illumina NovaSeq 6000 platform using PE250 sequencing mode with a target of 30,000 reads per sample. De-multiplexed reads were filtered (MaxEE = 5), trimmed (length 250), denoised, and merged using DADA2 (v1.36.0) (70) in RStudio (v4.5.0). Taxonomy was assigned using the AssignTaxonomy() function of DADA2 with the RefSeq v2 database (https://benjjneb.github.io/dada2/training.html). The resulting OTU table, taxonomic assignments, and sample metadata file were imported into R using Phyloseq (v1.52.0) (71).

Alpha diversity metrics (Shannon, Chao1, and Simpson) were calculated using the estimate_richness function of the Phyloseq package (v1.50.0). Beta diversity was assessed using two complementary approaches. First, PCoA based on the weighted UniFrac distance metric was used to assess variation in community structure. Permutational multivariate analysis of variance (PERMANOVA) was performed using the adonis2() function (1,000 permutations) with or without stratification by cage to test the effects of time and diet, and their interaction on community structure. Beta dispersion was calculated on the weighted UniFrac metric using the betadisper() function of vegan (v2.7.1). Genera significantly associated with variation in community structure were identified by regressing PCoA coordinates (axis 1 and axis 2) against the centered log-ratio (CLR) transformed taxonomic abundance data. Significant associations were identified based on regression results, with *P*-values derived from *t*-tests of slope coefficients for microbial genera. Second, distance-based redundancy analysis (db-RDA) was conducted on Bray-Curtis dissimilarity, constraining the ordination by the interaction between time and diet. Species loadings associated with the first two canonical axes were examined to determine the set of genera identified by both db-RDA and PCoA as driving change in community structure. Only genera that differed significantly across time by both methods and *post hoc* Wilcoxon rank sum tests were retained. For all measures, including alpha diversity and genus-level abundances, pairwise differences between time points or diet were assessed using the Wilcoxon rank sum test, with *P*-values adjusted for multiple testing with the Benjamini-Hochberg method (FDR < 0.05).

### Lipid droplet visualization and quantification

*C. albicans* cultures incubated for 24 h under anaerobic conditions were washed twice with 1× PBS and stained with 10 μM BODIPY (Thermo Fisher Scientific, Cat. No. D3922) for 30 min in the dark at room temperature. Cells were washed twice with 1× PBS to remove excess BODIPY and subsequently evaluated in a fluorescence microscope or by flow cytometry (BD LSRFortessa).

### RNA-seq analysis

The *C. albicans* reference strain and isogenic *sok1* deletion mutant were incubated under anaerobic conditions for 24 h in 10 mL of YNB broth supplemented with either dextrose or oleic acid. RNA purification was performed using the RiboPure RNA purification kit for yeast (Thermo Fisher Scientific, Cat. No. AM1926). Library preparation and sequencing were carried out by Novogene using poly-A enrichment, strand-specific library prep, and PE150 sequencing mode. Quality control, mapping, and differential gene expression analysis were carried out using protocols that the Perez lab has described before (34, 72) with Galaxy (https://usegalaxy.org/). Three biological replicates were included in the study. We obtained >35 million reads per sample with >95% of reads uniquely mapping to the *C. albicans* genome build 21 (http://www.candidagenome.org/). ORFs with a low number of reads (base mean < 150) were excluded from Gene Ontology analyses.

### Gene Ontology

The Gene Ontology term finder tool of the *Candida* Genome Database (http://www.candidagenome.org/) was used to identify overrepresented terms in the data sets.

### Western blot analysis

A *C. albicans* strain encoding Myc-tagged Cph2p was incubated under anaerobic conditions for 24 h in 20 mL of YNB broth supplemented with either dextrose or oleic acid. Cells were pelleted, washed with 1× Tris-buffered saline (TBS), and lysed as described (73). Crude cell extracts were resolved by electrophoresis in NuPAGE 4%–12% Bis-Tris mini gels (Thermo Fisher Scientific) and transferred to a PVDF membrane. The anti-Myc monoclonal antibody 9E10.3 (Thermo Fisher Scientific), an HRP-linked anti-mouse secondary antibody (Cytiva, Cat. No. NA931), and the SuperSignal West Femto substrate (Thermo Fisher Scientific) were used to detect the Myc-tagged protein. Alpha tubulin was detected with HRP-linked anti-tubulin alpha monoclonal antibody YL1/2 (Bio-Rad, Cat. No. MCA77P). A Bio-Rad ChemiDoc MP Imaging System was employed for imaging.

### Mucin binding assay

Porcine mucin II (Sigma, Cat. No. M2378) and III (Sigma, Cat. No. M1778) were fluorescently labeled with fluorescein-5-EX N-hydroxysuccinimide ester (Sigma, Cat. No. F9551) as described (74). *C. albicans* cultures incubated for 24 h under anaerobic conditions were washed twice with 1× PBS and stained with 50 μg/mL of fluorescein-labeled mucin for 60 min in the dark at room temperature. Mucin binding to *C. albicans* cells was evaluated by flow cytometry (BD LSRFortessa).

### Mannan staining

*C. albicans* cultures incubated for 24 h under anaerobic conditions were washed twice with 1× PBS and stained with 50 μg/mL concanavalin A conjugated to Alexa Fluor 488 (Thermo Fisher Scientific, Cat. No. C11252) for 30 min in the dark at room temperature. Cells were washed twice with 1× PBS to remove excess concanavalin A and subsequently evaluated by flow cytometry (BD LSRFortessa).

## Data Availability

Raw reads of all 16S rRNA sequencing samples have been deposited in the NCBI Sequence Read Archive (SRA) and can be accessed under BioProject PRJNA1277300. The RNA-Seq data have been deposited in the NCBI Gene Expression Omnibus (GEO) database under accession numbers GSE303952 and GSE303965. Code used to analyze the microbiome data are available at https://github.com/dmap02/perez_diet.

## AFTER EPUB

*[This article was published on 26 November 2025 with missing Author Contributions. The contributions were corrected in the current version, posted on 2 December 2025.]*

## SUPPLEMENTAL MATERIAL

Supplemental Figures mbio.02430-25-s0001.pdf Figures S1 to S3.

Table S1 mbio.02430-25-s0002.pdf Composition of the high-oleic safflower oil diet.

Table S2 mbio.02430-25-s0003.xlsx List of differentially expressed genes (OA vs. glucose).

Table S3 mbio.02430-25-s0004.xlsx List of differentially expressed genes (WT vs. *sok1*).

Table S4 mbio.02430-25-s0005.pdf *C. albicans* strains used in this study.

Table S5 mbio.02430-25-s0006.pdf Oligonucleotides used in this study.

ASM does not own the copyrights to Supplemental Material that may be linked to, or accessed through, an article. The authors have granted ASM a non-exclusive, world-wide license to publish the Supplemental Material files. Please contact the corresponding author directly for reuse.

## References

[1] Wu GD, Chen J, Hoffmann C, Bittinger K, Chen YY, Keilbaugh SA, Bewtra M, Knights D, Walters WA, Knight R, Sinha R, Gilroy E, Gupta K, et al.. 2011. Linking long-term dietary patterns with gut microbial enterotypes. Science 334:105–108. doi:10.1126/science.1208344 21885731 PMC3368382

[2] Wu M, McNulty NP, Rodionov DA, Khoroshkin MS, Griffin NW, Cheng J, Latreille P, Kerstetter RA, Terrapon N, Henrissat B, Osterman AL, Gordon JI. 2015. Genetic determinants of in vivo fitness and diet responsiveness in multiple human gut Bacteroides. Science 350:aac5992. doi:10.1126/science.aac5992 26430127 PMC4608238

[3] Pérez JC. 2019. Candida albicans dwelling in the mammalian gut. Curr Opin Microbiol 52:41–46. doi:10.1016/j.mib.2019.04.007 31132744

[4] d’Enfert C, Kaune A-K, Alaban L-R, Chakraborty S, Cole N, Delavy M, Kosmala D, Marsaux B, Fróis-Martins R, Morelli M, et al.. 2021. The impact of the fungus-host-microbiota interplay upon Candida albicans infections: current knowledge and new perspectives. FEMS Microbiol Rev 45:fuaa060. doi:10.1093/femsre/fuaa060 33232448 PMC8100220

[5] Schille TB, Sprague JL, Naglik JR, Brunke S, Hube B. 2025. Commensalism and pathogenesis of Candida albicans at the mucosal interface. Nat Rev Microbiol 23:525–540. doi:10.1038/s41579-025-01174-x 40247134

[6] Bensasson D, Dicks J, Ludwig JM, Bond CJ, Elliston A, Roberts IN, James SA. 2019. Diverse lineages of Candida albicans live on old oaks. Genetics 211:277–288. doi:10.1534/genetics.118.301482 30463870 PMC6325710

[7] Ropars J, Maufrais C, Diogo D, Marcet-Houben M, Perin A, Sertour N, Mosca K, Permal E, Laval G, Bouchier C, et al.. 2018. Gene flow contributes to diversification of the major fungal pathogen Candida albicans. Nat Commun 9:2253. doi:10.1038/s41467-018-04787-4 29884848 PMC5993739

[8] Acosta-Rodriguez EV, Rivino L, Geginat J, Jarrossay D, Gattorno M, Lanzavecchia A, Sallusto F, Napolitani G. 2007. Surface phenotype and antigenic specificity of human interleukin 17-producing T helper memory cells. Nat Immunol 8:639–646. doi:10.1038/ni1467 17486092

[9] Bacher P, Hohnstein T, Beerbaum E, Röcker M, Blango MG, Kaufmann S, Röhmel J, Eschenhagen P, Grehn C, Seidel K, et al.. 2019. Human anti-fungal Th17 immunity and pathology rely on cross-reactivity against Candida albicans. Cell 176:1340–1355. doi:10.1016/j.cell.2019.01.041 30799037

[10] Ost KS, Round JL. 2023. Commensal fungi in intestinal health and disease. Nat Rev Gastroenterol Hepatol 20:723–734. doi:10.1038/s41575-023-00816-w 37479823

[11] Pérez JC. 2021. Fungi of the human gut microbiota: roles and significance. Int J Med Microbiol 311:151490. doi:10.1016/j.ijmm.2021.151490 33676239

[12] Underhill DM, Braun J. 2022. Fungal microbiome in inflammatory bowel disease: a critical assessment. J Clin Invest 132:e155786. doi:10.1172/JCI155786 35229726 PMC8884899

[13] Li XV, Leonardi I, Putzel GG, Semon A, Fiers WD, Kusakabe T, Lin W-Y, Gao IH, Doron I, Gutierrez-Guerrero A, DeCelie MB, Carriche GM, Mesko M, Yang C, Naglik JR, Hube B, Scherl EJ, Iliev ID. 2022. Immune regulation by fungal strain diversity in inflammatory bowel disease. Nature 603:672–678. doi:10.1038/s41586-022-04502-w 35296857 PMC9166917

[14] Jiang L, Stärkel P, Fan J-G, Fouts DE, Bacher P, Schnabl B. 2021. The gut mycobiome: a novel player in chronic liver diseases. J Gastroenterol 56:1–11. doi:10.1007/s00535-020-01740-5 33151407 PMC7819863

[15] Chu H, Duan Y, Lang S, Jiang L, Wang Y, Llorente C, Liu J, Mogavero S, Bosques-Padilla F, Abraldes JG, Vargas V, Tu XM, Yang L, Hou X, Hube B, Stärkel P, Schnabl B. 2020. The Candida albicans exotoxin candidalysin promotes alcohol-associated liver disease. J Hepatol 72:391–400. doi:10.1016/j.jhep.2019.09.029 31606552 PMC7031049

[16] Böhm L, Torsin S, Tint SH, Eckstein MT, Ludwig T, Pérez JC. 2017. The yeast form of the fungus Candida albicans promotes persistence in the gut of gnotobiotic mice. PLoS Pathog 13:e1006699. doi:10.1371/journal.ppat.1006699 29069103 PMC5673237

[17] Tso GHW, Reales-Calderon JA, Tan ASM, Sem X, Le GTT, Tan TG, Lai GC, Srinivasan KG, Yurieva M, Liao W, Poidinger M, Zolezzi F, Rancati G, Pavelka N. 2018. Experimental evolution of a fungal pathogen into a gut symbiont. Science 362:589–595. doi:10.1126/science.aat0537 30385579

[18] Witchley JN, Penumetcha P, Abon NV, Woolford CA, Mitchell AP, Noble SM. 2019. Candida albicans morphogenesis programs control the balance between gut commensalism and invasive infection. Cell Host Microbe 25:432–443. doi:10.1016/j.chom.2019.02.008 30870623 PMC6581065

[19] Mitchell MK, Ellermann M. 2022. Long chain fatty acids and virulence repression in intestinal bacterial pathogens. Front Cell Infect Microbiol 12:928503. doi:10.3389/fcimb.2022.928503 35782143 PMC9247172

[20] Drake DR, Brogden KA, Dawson DV, Wertz PW. 2008. Thematic review series: skin lipids. antimicrobial lipids at the skin surface. J Lipid Res 49:4–11. doi:10.1194/jlr.R700016-JLR200 17906220

[21] Parsons JB, Yao J, Frank MW, Jackson P, Rock CO. 2012. Membrane disruption by antimicrobial fatty acids releases low-molecular-weight proteins from Staphylococcus aureus. J Bacteriol 194:5294–5304. doi:10.1128/JB.00743-12 22843840 PMC3457211

[22] Zhu M, Frank MW, Radka CD, Jeanfavre S, Xu J, Tse MW, Pacheco JA, Kim JS, Pierce K, Deik A, Hussain FA, Elsherbini J, Hussain S, Xulu N, Khan N, Pillay V, Mitchell CM, Dong KL, Ndung’u T, Clish CB, Rock CO, Blainey PC, Bloom SM, Kwon DS. 2024. Vaginal Lactobacillus fatty acid response mechanisms reveal a metabolite-targeted strategy for bacterial vaginosis treatment. Cell 187:5413–5430. doi:10.1016/j.cell.2024.07.029 39163861 PMC11429459

[23] Gunsalus KTW, Tornberg-Belanger SN, Matthan NR, Lichtenstein AH, Kumamoto CA. 2016. Manipulation of host diet to reduce gastrointestinal colonization by the opportunistic pathogen Candida albicans. mSphere 1:e00020-15. doi:10.1128/mSphere.00020-15 PMC4863630 27303684

[24] Kabara JJ, Swieczkowski DM, Conley AJ, Truant JP. 1972. Fatty acids and derivatives as antimicrobial agents. Antimicrob Agents Chemother 2:23–28. doi:10.1128/AAC.2.1.23 4670656 PMC444260

[25] McCrory C, Lenardon M, Traven A. 2024. Bacteria-derived short-chain fatty acids as potential regulators of fungal commensalism and pathogenesis. Trends Microbiol 32:1106–1118. doi:10.1016/j.tim.2024.04.004 38729839

[26] Liang S-H, Sircaik S, Dainis J, Kakade P, Penumutchu S, McDonough LD, Chen Y-H, Frazer C, Schille TB, Allert S, Elshafee O, Hänel M, Mogavero S, Vaishnava S, Cadwell K, Belenky P, Perez JC, Hube B, Ene IV, Bennett RJ. 2024. The hyphal-specific toxin candidalysin promotes fungal gut commensalism. Nature 627:620–627. doi:10.1038/s41586-024-07142-4 38448595 PMC11230112

[27] Yamaguchi N, Sonoyama K, Kikuchi H, Nagura T, Aritsuka T, Kawabata J. 2005. Gastric colonization of Candida albicans differs in mice fed commercial and purified diets. J Nutr 135:109–115. doi:10.1093/jn/135.1.109 15623841

[28] Hiltunen JK, Mursula AM, Rottensteiner H, Wierenga RK, Kastaniotis AJ, Gurvitz A. 2003. The biochemistry of peroxisomal beta-oxidation in the yeast Saccharomyces cerevisiae. FEMS Microbiol Rev 27:35–64. doi:10.1016/S0168-6445(03)00017-2 12697341

[29] Piekarska K, Mol E, van den Berg M, Hardy G, van den Burg J, van Roermund C, MacCallum D, Odds F, Distel B. 2006. Peroxisomal fatty acid beta-oxidation is not essential for virulence of Candida albicans. Eukaryot Cell 5:1847–1856. doi:10.1128/EC.00093-06 16963628 PMC1694795

[30] Song EM, Byeon JS, Lee SM, Yoo HJ, Kim SJ, Lee SH, Chang K, Hwang SW, Yang DH, Jeong JY. 2018. Fecal fatty acid profiling as a potential new screening biomarker in patients with colorectal cancer. Dig Dis Sci 63:1229–1236. doi:10.1007/s10620-018-4982-y 29516324

[31] Graef M. 2018. Lipid droplet-mediated lipid and protein homeostasis in budding yeast. FEBS Lett 592:1291–1303. doi:10.1002/1873-3468.12996 29397034 PMC5947121

[32] Martin S, Parton RG. 2006. Lipid droplets: a unified view of a dynamic organelle. Nat Rev Mol Cell Biol 7:373–378. doi:10.1038/nrm1912 16550215

[33] Savage HP, Bays DJ, Tiffany CR, Gonzalez MAF, Bejarano EJ, Carvalho TP, Luo Z, Masson HLP, Nguyen H, Santos RL, Reagan KL, Thompson GR, Bäumler AJ. 2024. Epithelial hypoxia maintains colonization resistance against Candida albicans. Cell Host Microbe 32:1103–1113. doi:10.1016/j.chom.2024.05.008 38838675 PMC11239274

[34] Del Olmo Toledo V, Puccinelli R, Fordyce PM, Pérez JC. 2018. Diversification of DNA binding specificities enabled SREBP transcription regulators to expand the repertoire of cellular functions that they govern in fungi. PLoS Genet 14:e1007884. doi:10.1371/journal.pgen.1007884 30596634 PMC6329520

[35] Pérez JC, Kumamoto CA, Johnson AD. 2013. Candida albicans commensalism and pathogenicity are intertwined traits directed by a tightly knit transcriptional regulatory circuit. PLoS Biol 11:e1001510. doi:10.1371/journal.pbio.1001510 23526879 PMC3601966

[36] Rosenbach A, Dignard D, Pierce JV, Whiteway M, Kumamoto CA. 2010. Adaptations of Candida albicans for growth in the mammalian intestinal tract. Eukaryot Cell 9:1075–1086. doi:10.1128/EC.00034-10 20435697 PMC2901676

[37] Lane S, Di Lena P, Tormanen K, Baldi P, Liu H. 2015. Function and regulation of Cph2 in Candida albicans. Eukaryot Cell 14:1114–1126. doi:10.1128/EC.00102-15 26342020 PMC4621314

[38] Chen C, Pande K, French SD, Tuch BB, Noble SM. 2011. An iron homeostasis regulatory circuit with reciprocal roles in Candida albicans commensalism and pathogenesis. Cell Host Microbe 10:118–135. doi:10.1016/j.chom.2011.07.005 21843869 PMC3165008

[39] Znaidi S, van Wijlick L, Hernández-Cervantes A, Sertour N, Desseyn J-L, Vincent F, Atanassova R, Gouyer V, Munro CA, Bachellier-Bassi S, Dalle F, Jouault T, Bougnoux M-E, d’Enfert C. 2018. Systematic gene overexpression in Candida albicans identifies a regulator of early adaptation to the mammalian gut. Cell Microbiol 20:e12890. doi:10.1111/cmi.12890 29998470 PMC6220992

[40] Bockmühl DP, Krishnamurthy S, Gerads M, Sonneborn A, Ernst JF. 2001. Distinct and redundant roles of the two protein kinase A isoforms Tpk1p and Tpk2p in morphogenesis and growth of Candida albicans. Mol Microbiol 42:1243–1257. doi:10.1046/j.1365-2958.2001.02688.x 11886556

[41] Kruppa M, Goins T, Cutler JE, Lowman D, Williams D, Chauhan N, Menon V, Singh P, Li D, Calderone R. 2003. The role of the Candida albicans histidine kinase [CHK1) gene in the regulation of cell wall mannan and glucan biosynthesis. FEMS Yeast Res 3:289–299. doi:10.1111/j.1567-1364.2003.tb00170.x 12689636

[42] Lu Y, Su C, Unoje O, Liu H. 2014. Quorum sensing controls hyphal initiation in Candida albicans through Ubr1-mediated protein degradation. Proc Natl Acad Sci USA 111:1975–1980. doi:10.1073/pnas.1318690111 24449897 PMC3918812

[43] Kramara J, Kim MJ, Ollinger TL, Ristow LC, Wakade RS, Zarnowski R, Wellington M, Andes DR, Mitchell AG, Krysan DJ. 2024. Systematic analysis of the Candida albicans kinome reveals environmentally contingent protein kinase-mediated regulation of filamentation and biofilm formation in vitro and in vivo. mBio 15:e01249-24. doi:10.1128/mbio.01249-24 38949302 PMC11323567

[44] Eckstein M-T, Moreno-Velásquez SD, Pérez JC. 2020. Gut bacteria shape intestinal microhabitats occupied by the fungus Candida albicans. Curr Biol 30:4799–4807. doi:10.1016/j.cub.2020.09.027 33035488

[45] Leonardi I, Gao IH, Lin W-Y, Allen M, Li XV, Fiers WD, De Celie MB, Putzel GG, Yantiss RK, Johncilla M, Colak D, Iliev ID. 2022. Mucosal fungi promote gut barrier function and social behavior via type 17 immunity. Cell 185:831–846. doi:10.1016/j.cell.2022.01.017 35176228 PMC8897247

[46] Nobile CJ, Mitchell AP. 2005. Regulation of cell-surface genes and biofilm formation by the C. albicans transcription factor Bcr1p. Curr Biol 15:1150–1155. doi:10.1016/j.cub.2005.05.047 15964282

[47] Mottola A, Ramírez-Zavala B, Hünniger K, Kurzai O, Morschhäuser J. 2021. The zinc cluster transcription factor Czf1 regulates cell wall architecture and integrity in Candida albicans. Mol Microbiol 116:483–497. doi:10.1111/mmi.14727 33860578

[48] Ichikawa Y, Bruno VM, Woolford CA, Kim H, Do E, Brewer GC, Mitchell AP. 2021. Environmentally contingent control of Candida albicans cell wall integrity by transcriptional regulator Cup9. Genetics 218:iyab075. doi:10.1093/genetics/iyab075 33989396 PMC8864738

[49] Askew C, Sellam A, Epp E, Mallick J, Hogues H, Mullick A, Nantel A, Whiteway M. 2011. The zinc cluster transcription factor Ahr1p directs Mcm1p regulation of Candida albicans adhesion. Mol Microbiol 79:940–953. doi:10.1111/j.1365-2958.2010.07504.x 21299649 PMC4092010

[50] Kankainen M, Paulin L, Tynkkynen S, von Ossowski I, Reunanen J, Partanen P, Satokari R, Vesterlund S, Hendrickx APA, Lebeer S, et al.. 2009. Comparative genomic analysis of Lactobacillus rhamnosus GG reveals pili containing a human- mucus binding protein. Proc Natl Acad Sci USA 106:17193–17198. doi:10.1073/pnas.0908876106 19805152 PMC2746127

[51] Martins M, Aymeric L, du Merle L, Danne C, Robbe-Masselot C, Trieu-Cuot P, Sansonetti P, Dramsi S. 2015. Streptococcus gallolyticus Pil3 pilus is required for adhesion to colonic mucus and for colonization of mouse distal colon. J Infect Dis 212:1646–1655. doi:10.1093/infdis/jiv307 26014801

[52] Stones DH, Krachler AM. 2016. Against the tide: the role of bacterial adhesion in host colonization. Biochem Soc Trans 44:1571–1580. doi:10.1042/BST20160186 27913666 PMC5134996

[53] Harayama T, Riezman H. 2018. Understanding the diversity of membrane lipid composition. Nat Rev Mol Cell Biol 19:281–296. doi:10.1038/nrm.2017.138 29410529

[54] Ernst R, Ejsing CS, Antonny B. 2016. Homeoviscous adaptation and the regulation of membrane lipids. J Mol Biol 428:4776–4791. doi:10.1016/j.jmb.2016.08.013 27534816

[55] Hidalgo MA, Carretta MD, Burgos RA. 2021. Long chain fatty acids as modulators of immune cells function: contribution of FFA1 and FFA4 receptors. Front Physiol 12:668330. doi:10.3389/fphys.2021.668330 34276398 PMC8280355

[56] Warden CH, Fisler JS. 2008. Comparisons of diets used in animal models of high-fat feeding. Cell Metab 7:277. doi:10.1016/j.cmet.2008.03.014 18396128 PMC2394560

[57] Thangamani S, Monasky R, Lee JK, Antharam V, HogenEsch H, Hazbun TR, Jin Y, Gu H, Guo GL. 2021. Bile acid regulates the colonization and dissemination of Candida albicans from the gastrointestinal tract by controlling host defense system and microbiota. J Fungi 7:1030. doi:10.3390/jof7121030 PMC8708873 34947012

[58] Ma J, Yang X, He J. 2024. Comprehensive gut microbiota composition and microbial interactions among the three age groups. PLoS One 19:e0305583. doi:10.1371/journal.pone.0305583 39423213 PMC11488730

[59] Santa-María C, López-Enríquez S, Montserrat-de la Paz S, Geniz I, Reyes-Quiroz ME, Moreno M, Palomares F, Sobrino F, Alba G. 2023. Update on anti-inflammatory molecular mechanisms induced by oleic acid. Nutrients 15:224. doi:10.3390/nu15010224 36615882 PMC9824542

[60] Lindemann-Perez E, Perez JC. 2024. Candida albicans natural diversity: a resource to dissect fungal commensalism and pathogenesis. Curr Opin Microbiol 80:102493. doi:10.1016/j.mib.2024.102493 38833793 PMC12743322

[61] Cravener MV, Do E, May G, Zarnowski R, Andes DR, McManus CJ, Mitchell AP. 2023. Reinforcement amid genetic diversity in the Candida albicans biofilm regulatory network. PLoS Pathog 19:e1011109. doi:10.1371/journal.ppat.1011109 36696432 PMC9901766

[62] Huang MY, Woolford CA, May G, McManus CJ, Mitchell AP. 2019. Circuit diversification in a biofilm regulatory network. PLoS Pathog 15:e1007787. doi:10.1371/journal.ppat.1007787 31116789 PMC6530872

[63] Wing A, Jeffery E, Church CD, Goodell J, Saavedra-Peña RDM, Saha M, Holtrup B, Voisin M, Alavi NS, Floody M, Wang Z, Zapadka TE, et al.. 2025. Dietary oleic acid drives obesogenic adipogenesis via modulation of LXRα signaling. Cell Rep 44:115527. doi:10.1016/j.celrep.2025.115527 40208790 PMC12073628

[64] García-Gamboa R, Kirchmayr MR, Gradilla-Hernández MS, Pérez-Brocal V, Moya A, González-Avila M. 2021. The intestinal mycobiota and its relationship with overweight, obesity and nutritional aspects. J Hum Nutr Diet 34:645–655. doi:10.1111/jhn.12864 33586805

[65] Owens SR, Greenson JK. 2007. The pathology of malabsorption: current concepts. Histopathology 50:64–82. doi:10.1111/j.1365-2559.2006.02547.x 17204022

[66] Gillum AM, Tsay EY, Kirsch DR. 1984. Isolation of the Candida albicans gene for orotidine-5’-phosphate decarboxylase by complementation of S. cerevisiae ura3 and E. coli pyrF mutations. Mol Gen Genet 198:179–182. doi:10.1007/BF00328721 6394964

[67] Nguyen N, Quail MMF, Hernday AD. 2017. An efficient, rapid, and recyclable system for CRISPR-mediated genome editing in Candida albicans. mSphere 2:e00149-17. doi:10.1128/mSphereDirect.00149-17 28497115 PMC5422035

[68] Gratacap RL, Rawls JF, Wheeler RT. 2013. Mucosal candidiasis elicits NF-κB activation, proinflammatory gene expression and localized neutrophilia in zebrafish. Dis Model Mech 6:1260–1270. doi:10.1242/dmm.012039 23720235 PMC3759345

[69] Ramírez-Zavala B, Krüger I, Dunker C, Jacobsen ID, Morschhäuser J. 2022. The protein kinase Ire1 has a Hac1-independent essential role in iron uptake and virulence of Candida albicans. PLoS Pathog 18:e1010283. doi:10.1371/journal.ppat.1010283 35108336 PMC8846550

[70] Callahan BJ, McMurdie PJ, Rosen MJ, Han AW, Johnson AJA, Holmes SP. 2016. DADA2: high-resolution sample inference from Illumina amplicon data. Nat Methods 13:581–583. doi:10.1038/nmeth.3869 27214047 PMC4927377

[71] McMurdie PJ, Holmes S. 2013. Phyloseq: an R package for reproducible interactive analysis and graphics of microbiome census data. PLoS One 8:e61217. doi:10.1371/journal.pone.0061217 23630581 PMC3632530

[72] Böhm L, Muralidhara P, Pérez JC. 2016. A Candida albicans regulator of disseminated infection operates primarily as a repressor and governs cell surface remodeling. Mol Microbiol 100:328–344. doi:10.1111/mmi.13320 26700268

[73] Moreno-Velásquez SD, Tint SH, Del Olmo Toledo V, Torsin S, De S, Pérez JC. 2020. The regulatory proteins Rtg1/3 govern sphingolipid homeostasis in the human-associated yeast Candida albicans. Cell Rep 30:620–629. doi:10.1016/j.celrep.2019.12.022 31968241

[74] de Repentigny L, Aumont F, Bernard K, Belhumeur P. 2000. Characterization of binding of Candida albicans to small intestinal mucin and its role in adherence to mucosal epithelial cells. Infect Immun 68:3172–3179. doi:10.1128/IAI.68.6.3172-3179.2000 10816460 PMC97555

