# Supplementary material for: Diet-responsive genetic determinants of intestinal colonization in the yeast Candida albicans
Source: mBio. 2025 Nov 26;17(1):e02430-25. doi: 10.1128/mbio.02430-25 (PMC12802223; doi:10.1128/mbio.02430-25)
Supplement: Supplemental Figures — Figures S1 to S3. [file mbio.02430-25-s0001.pdf]

Supplemental Material

Diet-responsive genetic determinants of intestinal colonization in the yeast *Candida albicans*
Musfirat Shubaita, Mazen Oneissi, Elena Lindemann-Pérez, Cecilia Fadhel Alvarez, Anne-Marie Krachler, Diana M. Proctor, and J. Christian Pérez

Supplemental Figure Legends

Figure S1. Effects of diet on gut bacterial diversity, composition, and community structure. (A) Additional metrics of alpha diversity. Plotted are Observed richness, Simpson index, and Chao1. *P* values shown only if significant. (B) Top 20 taxa contributing to variation along the first and second db-RDA axes which account for ~17% of the variance. Bar plots display the top 20 genera with the highest absolute loadings on CAP1 (left, orange) and CAP2 (right, blue) from the db-RDA. Bars represent the magnitude and direction of each genus contribution to the corresponding canonical axis. All taxa are classified at the genus level. (C) Taxa abundance comparisons between PD and HOA diets. Plotted are centered log-ratios (CLR). The taxa included here are those described in Fig. 2E. Statistical analysis using Wilcoxon rank sum test and adjusted for multiple comparisons using Benjamini-Hochberg to control the False Discovery Rate (FDR) at 5%. *, $P < 0.05$

Figure S2. *C. albicans* WT and *fox2*Δ/Δ growth and viability. (A) Schematic of the *FOX2* locus. (B) Agarose gel analysis of PCR products amplified with oligos targeting *FOX2* internal region. (C) Spot assays with the indicated strains on YNB agar supplemented with either glucose or oleic acid as the only carbon source. Agar plates were incubated at 30°C in normal air. (D and E). Cell viability under anaerobic conditions. The indicated strains were inoculated in either THB (D) or YNB broth (E) supplemented with glucose and/or oleic acid. At the indicated time points, cultures were sampled, diluted and plated on YPD agar for colony enumeration. YPD agar plates were incubated at 30°C in normal air. The experiment was repeated 4 times. LOD, limit of detection. (F) No change in cell surface mannan in *fox2*Δ/Δ strain. Concanavalin A conjugated to Alexa Fluor 488 was incubated with the indicated *C. albicans* strains which were grown anaerobically in YNB broth containing oleic acid. Flow cytometry was used for quantification. 30,000 single cells were examined per strain per experiment. Each dot represents the mean fluorescence of an independent experiment. (G) *C. albicans* binding to mucin is *FOX2*-independent. Fluorescein-labeled mucin type II was incubated with the indicated *C. albicans* strains which were grown anaerobically in YNB broth containing oleic acid. Flow cytometry was used for quantification. 30,000 single cells were examined per strain per experiment. Each dot represents an independent experiment.

Figure S3. Effect of fatty acids on *C. albicans* morphology, cell surface mannan exposure, and mucin binding. (A) *C. albicans* cell morphology quantification under anaerobic conditions. Wild-type *C. albicans* cells were inoculated in YNB broth supplemented with either glucose or oleic acid. At the indicated time points, aliquots from each culture were taken and evaluated under a microscope. At least 2,000 cells were scored per time point per condition. Filamenting cells were defined as those with length >2X width. Each circle represents the mean of one independent experiment. LOD, limit of detection. (B) *C. albicans* binding to mucin. Fluorescein-labeled mucin type II was incubated with wild-type *C. albicans* cells grown anaerobically in

YNB broth ± oleic acid. Flow cytometry was used for quantification. 30,000 single cells were examined per strain per experiment. Each dot represents an independent experiment. Mean ± s.e.m. are indicated. (C) Cell surface mannan quantification. Concanavalin A conjugated to Alexa Fluor 488 was incubated with wild-type *C. albicans* which had been grown anaerobically in YNB broth ± oleic acid. 30,000 single cells were examined per strain per experiment by flow cytometry. Each dot represents the mean fluorescence of an independent experiment. Mean ± s.e.m. are indicated. (D) Total fungal load of the mouse intestinal colonization experiments shown in Figs. 5H and 5I. Each circle represents an individual mouse. Median and interquartile range are plotted. Statistical analysis in (B) and (C) using the unpaired *t*-test (two-tailed); in (D) with the Mann-Whitney U test.

Fig. S1

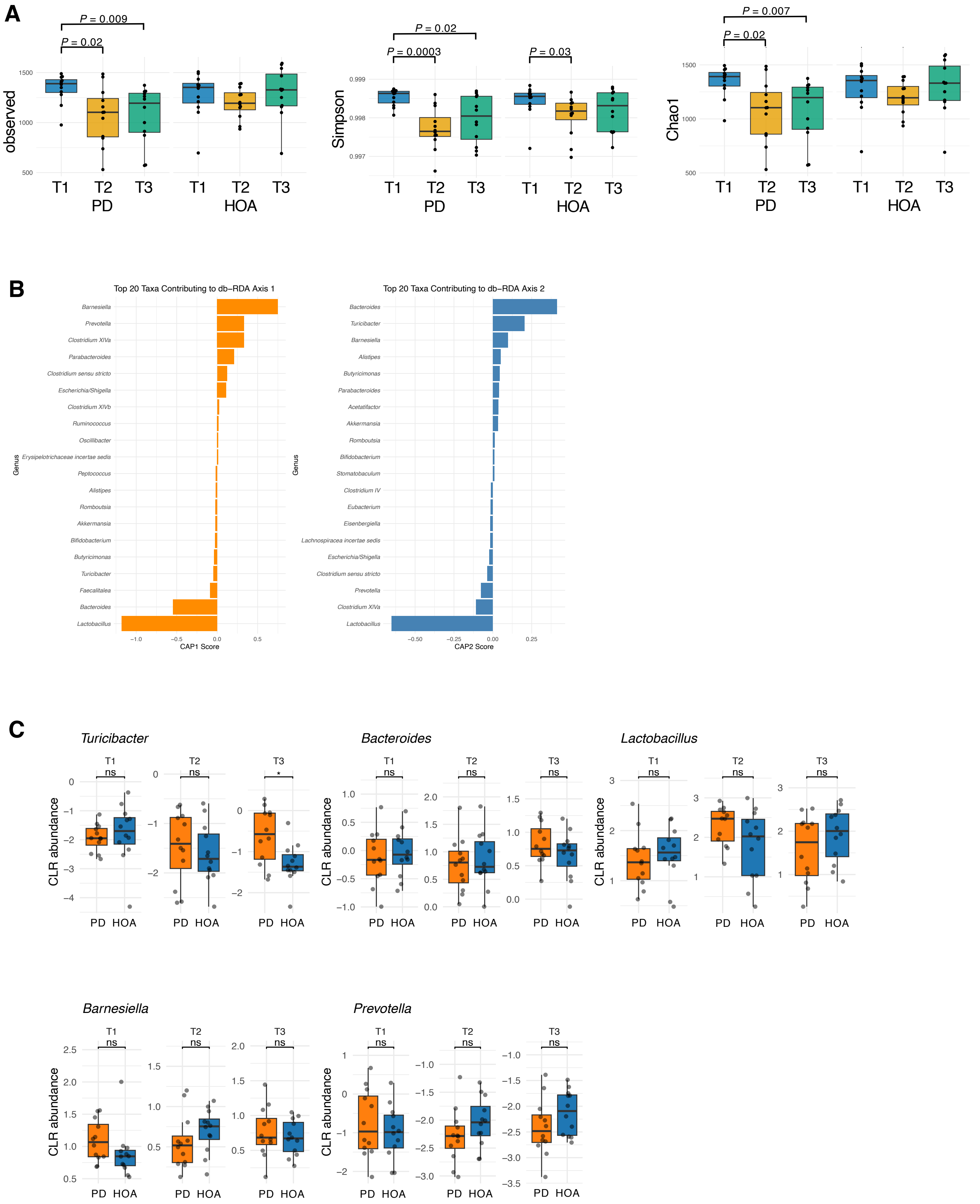
A
P = 0.009
P = 0.02
observed
T1 T2 T3 T1 T2 T3
PD HOA
P = 0.02
P = 0.0003
P = 0.03
Simpson
P = 0.007
P = 0.02
Chao1
B
Top 20 Taxa Contributing to db−RDA Axis 1
Top 20 Taxa Contributing to db−RDA Axis 2
Genus
CAP1 Score
CAP2 Score
Barnesiella
Prevotella
Clostridium XlVa
Parabacteroides
Clostridium sensu stricto
Escherichia/Shigella
Clostridium XlVb
Ruminococcus
Oscillibacter
Erysipelotrichaceae incertae sedis
Peptococcus
Alistipes
Romboutsia
Akkermansia
Bifidobacterium
Butyricimonas
Turicibacter
Faecalitalea
Bacteroides
Lactobacillus
Acetatifactor
Stomatobaculum
Clostridium IV
Eubacterium
Eisenbergiella
Lachnospiracea incertae sedis
C
Turicibacter
Bacteroides
Lactobacillus
Barnesiella
Prevotella
T1 T2 T3
ns
*
CLR abundance
PD HOA

Fig. S2

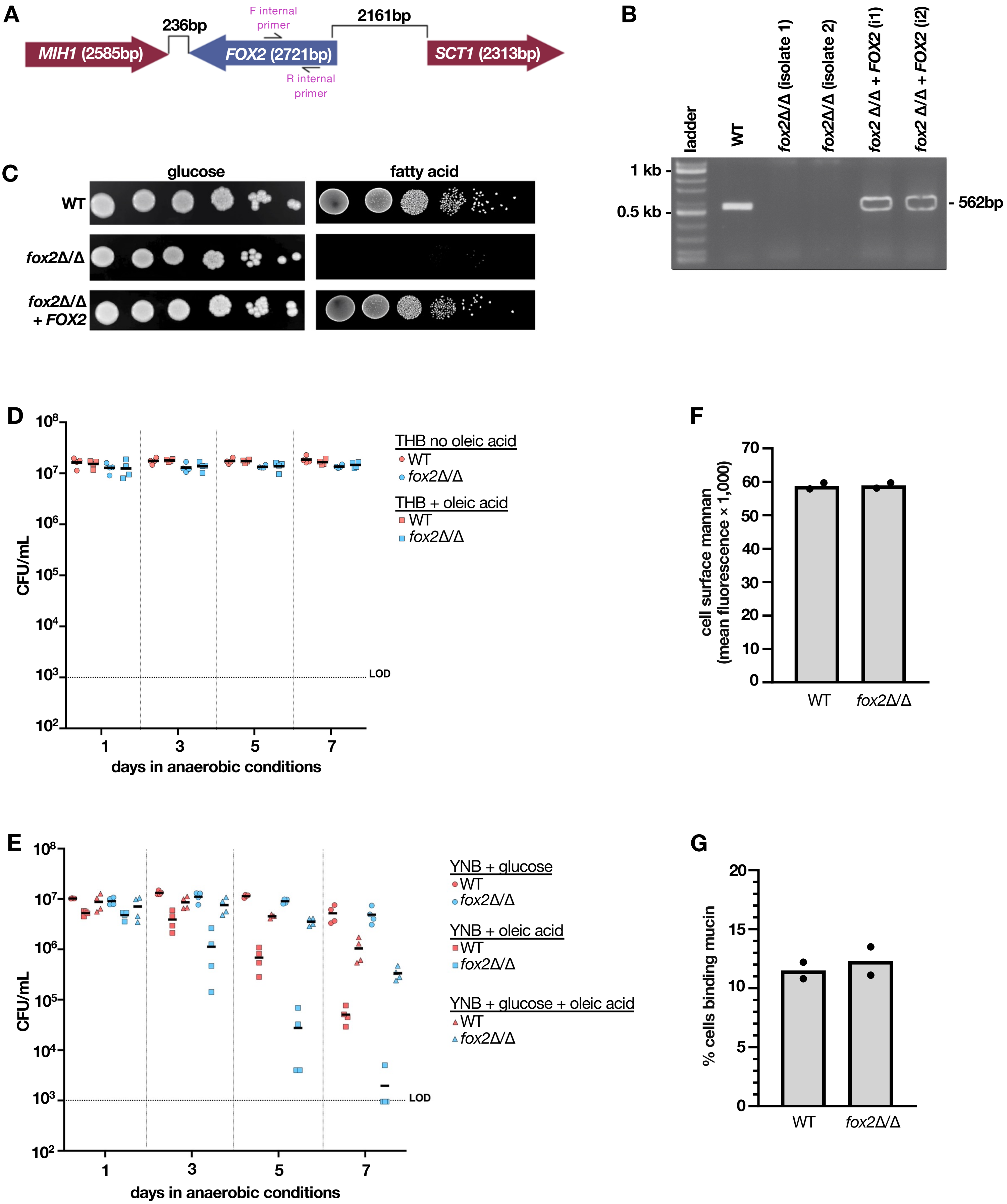
A
236bp
F internal primer
2161bp
MIH1 (2585bp)
FOX2 (2721bp)
SCT1 (2313bp)
R internal primer
B
ladder
WT
fox2Δ/Δ (isolate 1)
fox2Δ/Δ (isolate 2)
fox2 Δ/Δ + FOX2 (i1)
fox2 Δ/Δ + FOX2 (i2)
1 kb
0.5 kb
562bp
C
glucose
fatty acid
WT
fox2Δ/Δ
fox2Δ/Δ + FOX2
D
CFU/mL
LOD
days in anaerobic conditions
THB no oleic acid
WT
fox2Δ/Δ
THB + oleic acid
WT
fox2Δ/Δ
E
CFU/mL
LOD
days in anaerobic conditions
YNB + glucose
WT
fox2Δ/Δ
YNB + oleic acid
WT
fox2Δ/Δ
YNB + glucose + oleic acid
WT
fox2Δ/Δ
F
cell surface mannan (mean fluorescence × 1,000)
WT
fox2Δ/Δ
G
% cells binding mucin
WT
fox2Δ/Δ

Fig. S3

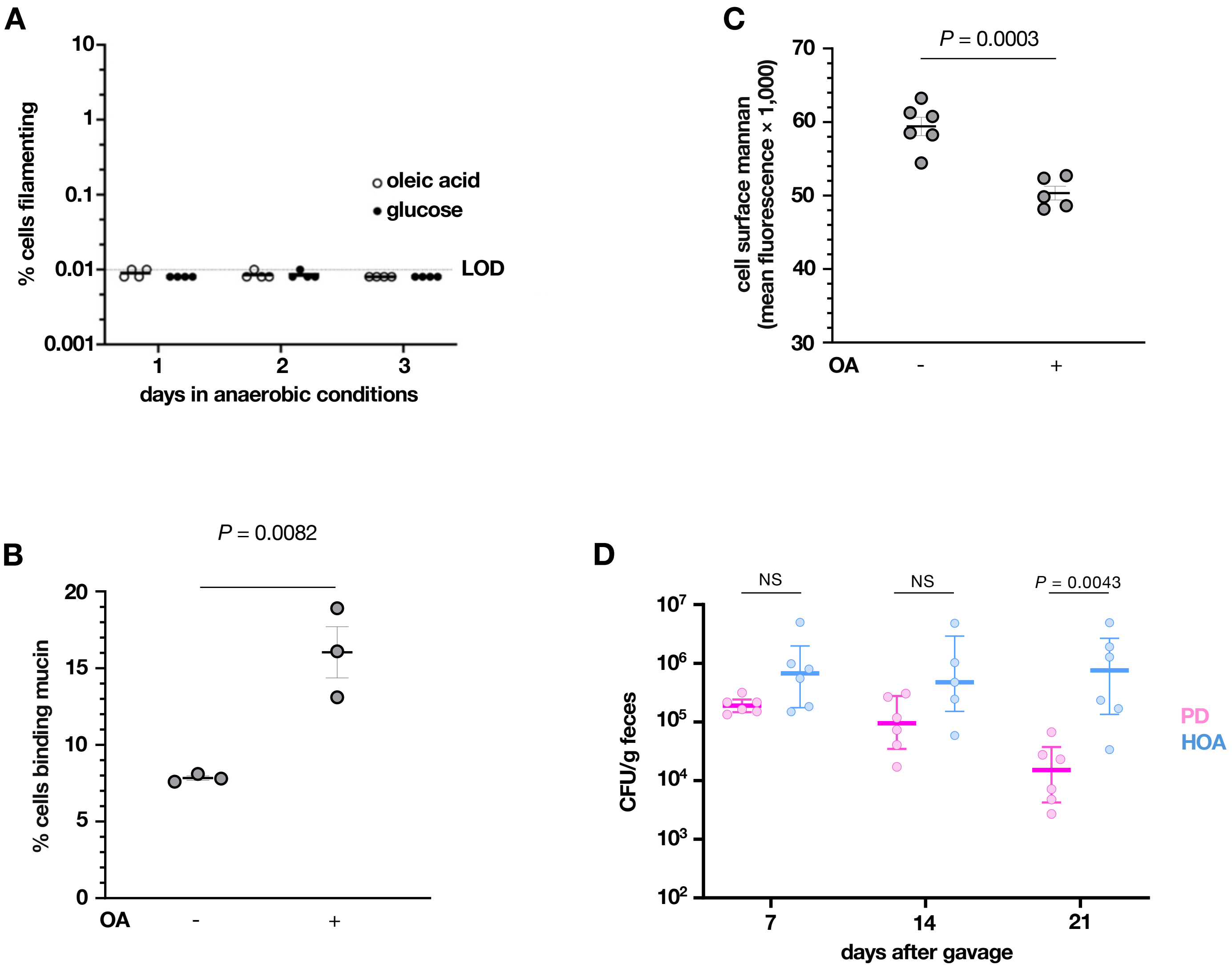
A
% cells filamenting
10
1
0.1
0.01
0.001
oleic acid
glucose
LOD
1
2
3
days in anaerobic conditions
C
P = 0.0003
cell surface mannan
(mean fluorescence × 1,000)
70
60
50
40
30
OA
-
+
B
P = 0.0082
% cells binding mucin
20
15
10
5
0
OA
-
+
D
NS
NS
P = 0.0043
CFU/g feces
10^7
10^6
10^5
10^4
10^3
10^2
PD
HOA
7
14
21
days after gavage
