# Supplementary material for: Diet-responsive genetic determinants of intestinal colonization in the yeast Candida albicans
Source: mBio. 2025 Nov 26;17(1):e02430-25. doi: 10.1128/mbio.02430-25 (PMC12802223; doi:10.1128/mbio.02430-25)
Supplement: Table S1 — Composition of the high-oleic safflower oil diet. [file mbio.02430-25-s0002.pdf]

Table S1. Composition of HOA diet

**Product**	**gram**	**kcal**
Protein	24.3	20
Carbohydrate	41	34
Fat	24.2	45
Total		*100*
kcal/gm	4.79	
**Ingredient**		
Casein, 30 Mesh	200	800
L-Cystine	3	12
Corn Starch	100.5	402
Maltodextrin	132	528
Sucrose	100	400
Cellulose, BW200	50	0
Safflower Oil, High Oleic	202	1818
tBHQ	0.0393	0
AIN-93G Salts	35	0
V10037	10	40
Choline Bitartrate	2.5	0
FD&C Yellow Dye #5	0.025	0
FD&C Blue Dye #1	0.025	0
Total	*835.089*	*4000*

Formulated by Research Diets, Inc. (Cat. No. D21040907)
