# Supplementary material for: Diet-responsive genetic determinants of intestinal colonization in the yeast Candida albicans
Source: mBio. 2025 Nov 26;17(1):e02430-25. doi: 10.1128/mbio.02430-25 (PMC12802223; doi:10.1128/mbio.02430-25)
Supplement: Table S4 — C. albicans strains used in this study. [file mbio.02430-25-s0005.pdf]

Table S4. ***Candida albicans*** **strains used in this study**

Strain	Genotype	Source
SC5314	wild-type prototroph	[66]
AHY940	*leu2* Δ/*LEU2*	[67]
JCP_1420	*leu2* Δ/*LEU2 fox2* Δ/*fox2* Δ	This work
JCP_1441	*leu2* Δ/*LEU2 fox2* Δ/*fox2* Δ + *FOX2*	This work
JCP_960	*ADE2* /*ade2* Δ::*CPH2p* -myc-*CPH2* -URA3	[37]
JCP_1524	*chk1* Δ/*chk1* Δ	[69]
JCP_1525	*sok1* Δ/*sok1* Δ	[69]
JCP_1526	*sok1* Δ/*sok1* Δ + *SOK1*	[69]
JCP_1423	SC5314 *ENO1* p-dTomato	This work
JCP_1321	*leu2* Δ/*LEU2* *ENO1* p-dTomato	This work
