# Supplementary material for: Diet-responsive genetic determinants of intestinal colonization in the yeast Candida albicans
Source: mBio. 2025 Nov 26;17(1):e02430-25. doi: 10.1128/mbio.02430-25 (PMC12802223; doi:10.1128/mbio.02430-25)
Supplement: Table S5 — Oligonucleotides used in this study. [file mbio.02430-25-s0006.pdf]

Table S5. Oligos used in this study

Name	Description	Sequence (5' to 3')
JCP_4076	Univ_Frag_A_gRNA (AHO1096)	GACGGCACGGCCACGCGTTTAAACCGCC
JCP_4077	gRNA Fragment B (AHO1097)	CCCGCCAGGCGCTGGGGTTTAAACACCG
JCP_4079	Frag C (AHO1237)	AGGTGATGCTGAAGCTATTGAAG
JCP_4080	Frag C for LEUpOUT (AHO1238)	TGTATTTTGTTTTAAAATTTTAGTGACTGTTTC
JCP_4550	FOX2_Forward_Repair	TTCTATTTTATTAACTTCAAACATAATACTTTCCTCCCCCCCCCCATTAGG<br>ACGCAGTAAAATTGCCATTTAGTTTTTTATATTATTTTGTTTTGTTAGT
JCP_4551	FOX2_Reverse_Repair	ACTAACAAAACAAAATAATATAAAAAACTAAATGGCAATTTTACTGCGTCC<br>TAATGGGGGGGGGGAGGAAAGTATTATGTTTGAAGTTAATAAAATAGAA
JCP_4553	FOX2_KIgRNA	CGTAAACTATTTTTAATTTGCTTTCCTCCCCCCCCCCATTGTTTTAGAGCT<br>AGAAATAGC
JCP_4560	Fox2_internal check_F2	TGCCTCCCAAGCTGAAGAAA
JCP_4561	Fox2_internal check_R2	AGGTCATGGCAGTTTCAGCA
JCP_4576	FOX2_KOgRNA	CGTAAACTATTTTTAATTTGGTCACCAGTCAAACGGTACAGTTTTAGAGCT<br>AGAAATAGC
JCP_4558	FOX2_Check_Up+Downstream_F	TCTATGTCGTTGTGTTATGTTAAAGTGGA
JCP_4580	FOX2_Check_Up+Downstream_R	AAGCTGATTAGATTCAAATTGCATAAACG
JCP_4699	FOX2_internal check_F1	ACGTAACTTCAACCCAATGT
JCP_4700	FOX2_internal check_R1	GTCATCGCTTTCTTTCCAAG
